# Supplementary material for: Expression Pattern and Functional Analysis of MebHLH149 Gene in Response to Cassava Bacterial Blight
Source: Plants (Basel). 2024 Aug 30;13(17):2422. doi: 10.3390/plants13172422 (PMC11397265; doi:10.3390/plants13172422)
Supplement: Supplementary file 1 [file plants-13-02422-s001.zip › Figure S1.pdf]

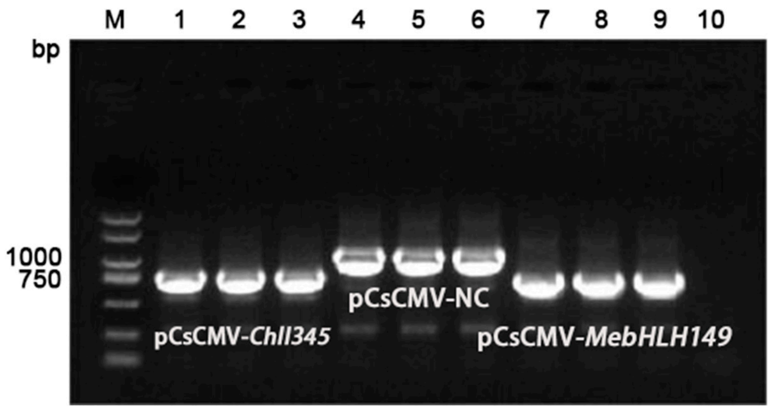

Figure S1. Gel electrophoresis of *MebHLH149* in cassava silenced plants: The DNA ladder used was the DL2000 bp marker (M), serves as a reference for estimating size of the DNA fragment. Lanes 1-3: Represent the positive control *pCsCMV-ChlI345*; Lanes 4-6: Represent the negative control *pCsCMV-NC* ; 7-8: Represent the experimental group *pCsCMV-MebHLH149*. Lane 10 is the negative control, using double-distilled H_2O (ddH_2O) instead of DNA.
