# Supplementary material for: Expression Pattern and Functional Analysis of MebHLH149 Gene in Response to Cassava Bacterial Blight
Source: Plants (Basel). 2024 Aug 30;13(17):2422. doi: 10.3390/plants13172422 (PMC11397265; doi:10.3390/plants13172422)
Supplement: Supplementary file 1 [file plants-13-02422-s001.zip › Figure S2.pdf]

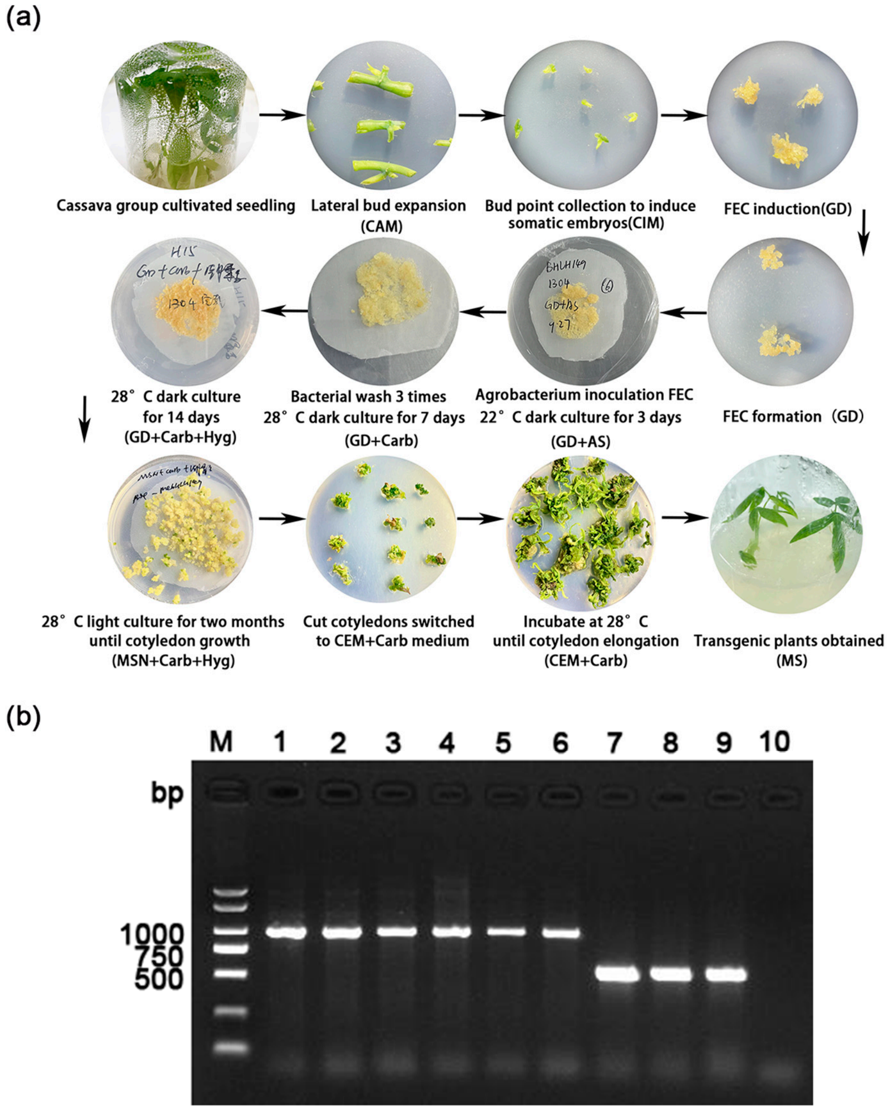

Figure S2. Overexpression of the *MebHLH149* gene in cassava: (a) Workflow for cassava FEC infestation with the *MebHLH149* gene . FEC: friable embryogenic callus; GD: Gresshof and Doy Basal Medium ; AS: Acetosyringone; Carb: carbenicillin; Hyg:hygromycin ; CAM: Callus induction medium; CIM: Callus induction medium, a callus induction medium. The plant growth regulators added are different. 6-BA (6-Benzyladenine) is added to CAM, and picloram is added to CIM; MSN: It has more plant growth regulator NAA than the MS medium. ; MS: Murashige and Skoog ;(b) Electrophoresis analysis of transgenic seedlings. In (b), the DNA ladder used was the DL2000 bp marker (M), serves as a reference for estimating size of the DNA fragment. Lanes 1-6 show results for transgenic plants with overexpression of *MebHLH149* gene. Lanes 7 and 9 correspond to empty vector controls, which were not modified with the *MebHLH149* gene. Lane 10 is the negative control, using double-distilled H_2O (ddH_2O) instead of DNA.
