# Supplementary material for: Expression Pattern and Functional Analysis of MebHLH149 Gene in Response to Cassava Bacterial Blight
Source: Plants (Basel). 2024 Aug 30;13(17):2422. doi: 10.3390/plants13172422 (PMC11397265; doi:10.3390/plants13172422)
Supplement: Supplementary file 1 [file plants-13-02422-s001.zip › Gel electrophoresis raw image.pdf]

About the agarose gel electrophoreogram of this study

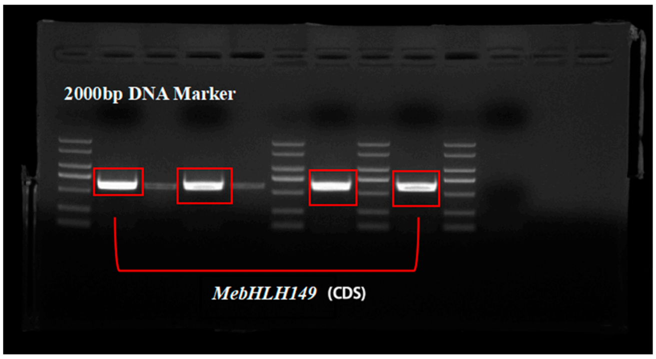

Gel electrophoresis of *bHLH149* CDS amplification.

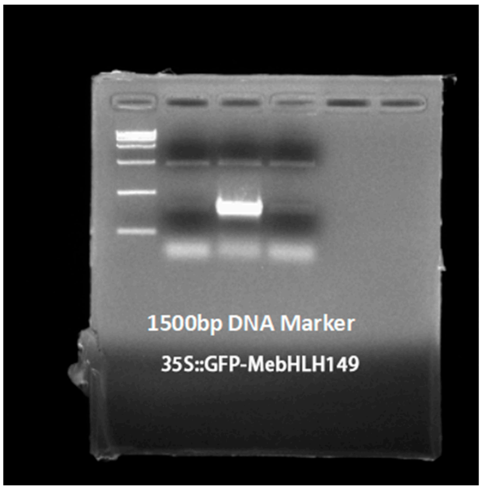

Gel electrophoresis of the *MebHLH149* fragment used for Subcellular amplification.

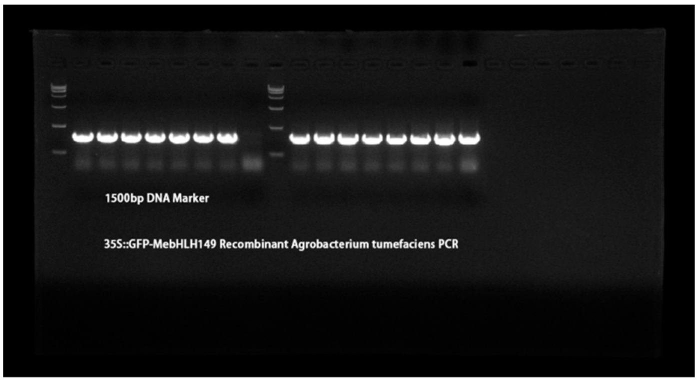

Gel electrophoresis of *MebHLH149* fragment used for Subcellular localisation.

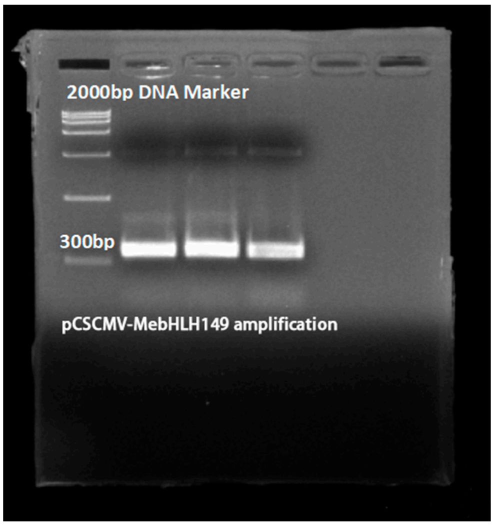

Gel electrophoresis of the MebHLH149 amplified fragment used for VIGS.

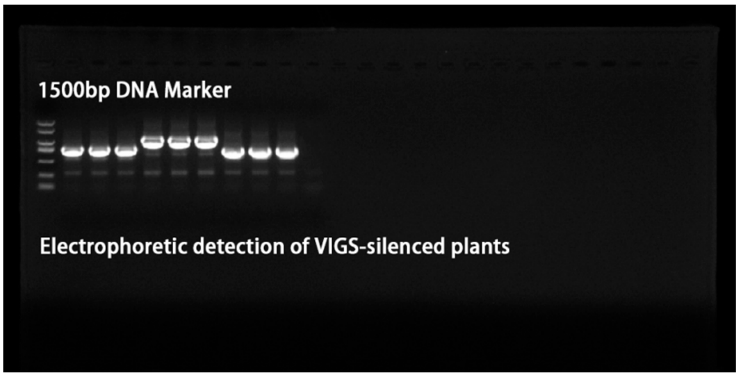

Gel electrophoresis of *MebHLH149* in cassava silenced plants.

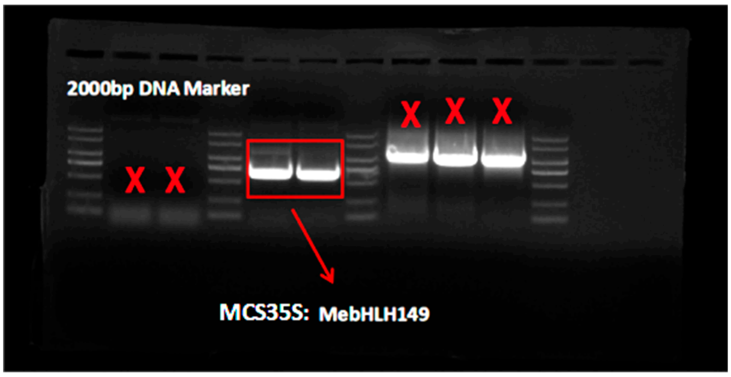

Gel electrophoresis of *bHLH149* amplification in cassava overexpressing plants.

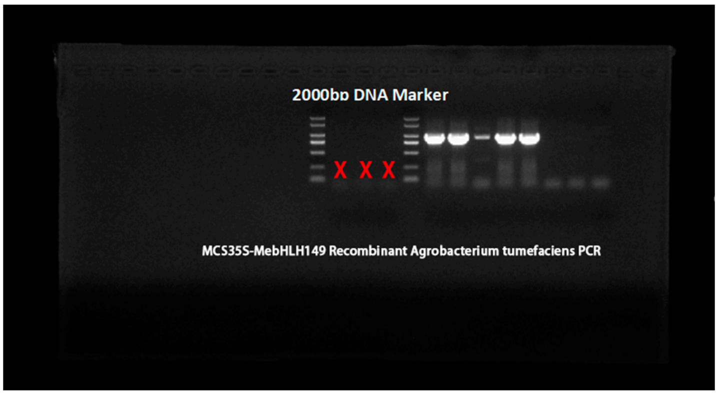

Electrophoretic visualization of *MebHLH149* expressed in *Agrobacterium tumefaciens.*

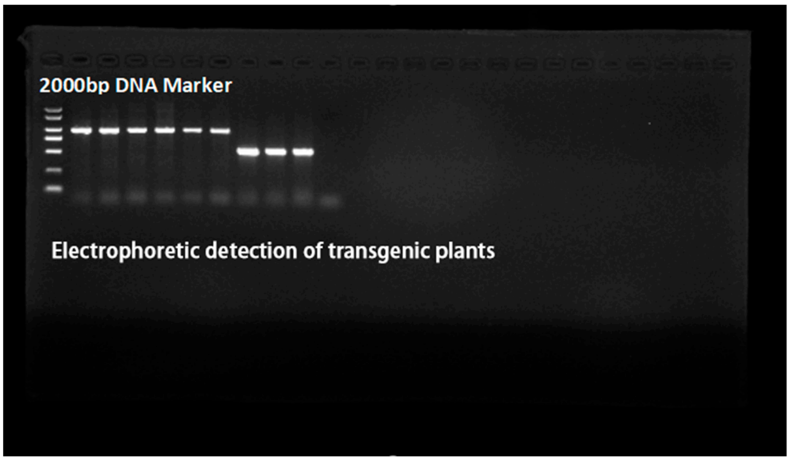

Electrophoretic visualization of gene amplifications for Identification of transgenic positive plants.

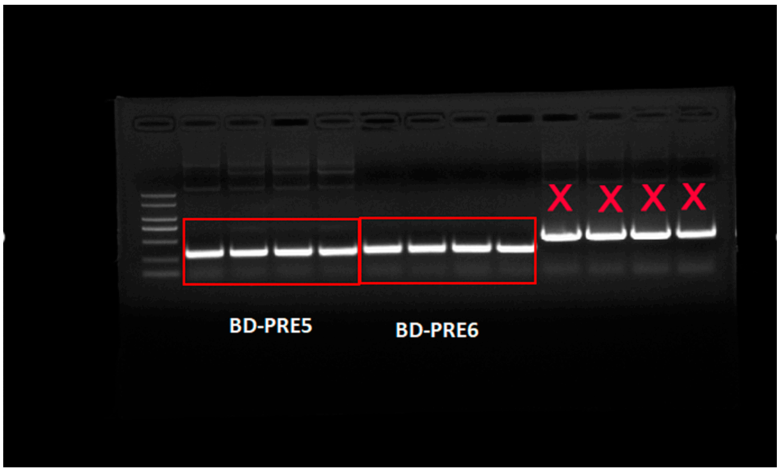

Gel electrophoresis of *MePRE5*, *MePRE6* amplification for yeast two-hybrid (Y2H) system.

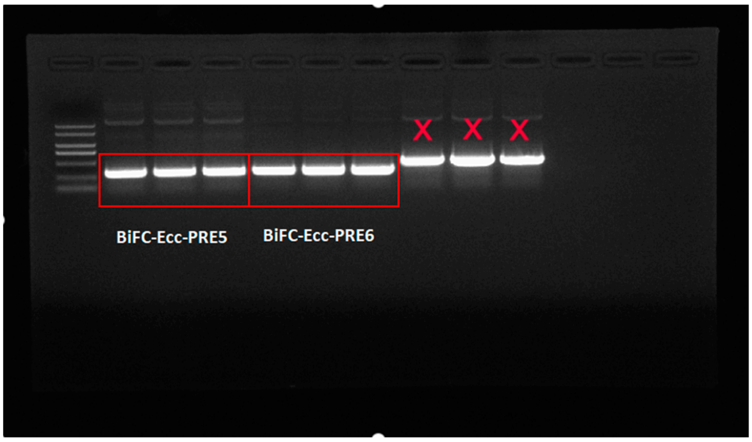

Gel electrophoresis of *MePRE5*, *MePRE6* amplification for bimolecular fluorescence complementation (BiFC).

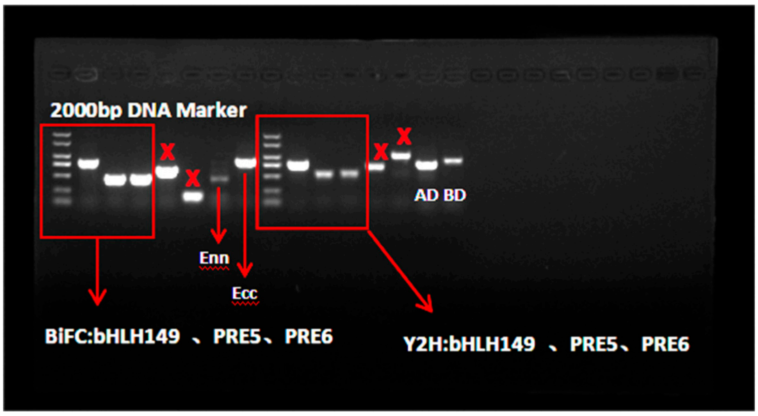

Gel electrophoresis of *MebHLH149*, *MePRE5*, *MePRE6* for bimolecular fluorescent complementation and yeast two-hybrid system analyses.
