# Supplementary material for: Expression Pattern and Functional Analysis of MebHLH149 Gene in Response to Cassava Bacterial Blight
Source: Plants (Basel). 2024 Aug 30;13(17):2422. doi: 10.3390/plants13172422 (PMC11397265; doi:10.3390/plants13172422)
Supplement: Supplementary file 1 [file plants-13-02422-s001.zip › Tables.pdf]

Table S1. Primers used for quantitative real-time PCR.

Gene	Primer	Sequence
MebHLH149	QbHLH149-F	AAGGAGAGCAGTAGGTTA
	QbHLH149-R	ATTAGTAAACGATGTCTTCTG
MebHLH147-1	QMebHLH147-1F	TGAAAGGGAAGAGTTTAC
	QMebHLH147-1R	TAGAATAACAGGCAATGG
MebHLH147-2	QMebHLH147-2F	ATGGCATCGTTGATCTCA
	QMebHLH147-2R	TCTTCGCTTCTTCCTGTG
MebHLH147-3	QMebHLH147-3F	CATCCTGTAACTAATTCCAACTTC
	QMebHLH147-3R	GCGTGGTCTTGAATCTGA
MebHLH153	QMebHLH153-F	GGAATACAGTCTGAGAAG
	QMebHLH153-R	GGAGAAGTAGTCTTGATG
MebHLH106	QMebHLH106-F	CCTCACAAATTCCAGGGCTC
	QMebHLH106-F	CAGGCAAAAGGTCAGAGCGG
MebHLH162	QMebHLH162-F	GTGTTCCATACAATCCAT
	QMebHLH162-R	CAACTCCAGTCAATCAAT
MebHLH61	QMebHLH61-F	GCTCCGTGATGTTACCAA
	QMebHLH61-R	GCTGCTTGAGAAGTTCCTAT
MeCAD8	QMeCAD8-F	GCAGGGATTGTGGGTTTAG
	QMeCAD8-R	AACCAGGAAAGCATCAGCA
MeCAD15	QMeCAD15-F	GGGAATGTCACCAGAACAA
	QMeCAD15-R	TACTCCTCCAAGCCCTAAT
MeCAD16	QMeCAD16-F	GATGGGATGTCACCAGAAC
	QMeCAD16-R	CTCCTCTTAGCCCACTCTT
MePOD12	QMePOD-F	GAGACGGTGTCGTATTTGCTCA
	QMePOD-R	GGTTGGATAAAGACGGTCAGTG
MeEDS1	QMeEDS1-F	AGAGCCTCGGGATCAGAGAA
	QMeEDS1-R	TTCCCTGCGTATTTGTGGGT
MeMYC2	QMeMYC2-F	ACCTCGGGTCATCTTCAATG

	QMeMYC2-R	GGGTCATTAACATCTGGGGACA
MeNCED6	QMeNCED6-F	CCGCATTGGCTAGGTCTCTT
	QMeNCED6-R	CCAGTGCCTCGTGATGTGTT
MeGST23	QMeGST23-F	AGAGTCCCTTGCTGTTGCTG
	QMeGST23-R	AGAGACTCCGCCACAGGATT
MePPE5	QMePPE5-F	CTAGCAGAAGGTCCAGGCAG
	QMePPE5-R	GGTGCGCCTATCACGAATCT
MePPE6	QMePPE6-F	TCGTTCGAGACAGACACCAG
	QMePPE6-R	CACCTTTGAAGCCGAGACCT

Table S2. Primers used for gene fragment ampliifications and vector constructions.

Gene	Primer	Sequence	Vector
MebHLH149	MebHLH149-F	ATGGCTTCGTTGATCTCAAGC	CDS
	MebHLH149-R	TTAGCTTGTGGGCCTAACCG	
MebHLH149	MebHLH149 -SubN-F	agtggtctctgtccagtcctATGGCTTCG TTGATCTCAAG	pNC-Green-Sub N
	MebHLH149 -SubN-R	ggtctcagcagaccacaagtGCTTGTGG GCCTAACCGG	
MebHLH149	MebHLH149 -VIGS-F	agtggtctctgtccagtcctATGGCTTCG TTGATCTCAAGCCT	PNC-CsCMV
	MebHLH149 -VIGS-R	ggtctcagcagaccacaagtCGCGCGG CTCCACCGAGT	
MebHLH149	MebHLH149 -MCS35S-F	agtggtctctgtccagtcctATGGCTTCG TTGATCTCAAGC	PNC-Cam1304- MCS35S
	MebHLH149 -MCS35S-R	ggtctcagcagaccacaagtTTAGCTTG TGGGCCTAACCG	
MebHLH149	MebHLH149 -AD-F	As above overexpression amplification primer F	PNC-GADT7
	MebHLH149 -AD-R	As above overexpression amplification primer R	
MePPE5	MePRE5 -BD-F	agtggtctctgtccagtcctATGTCTAGC AGAAGGTCC	PNC-GBKT7
	MePRE5 -BD-R	ggtctcagcagaccacaagtTTACATGA TTAAACTCCTTATTA	
MePPE6	MePRE6 -BD-F	agtggtctctgtccagtcctATGTCTAGC AGAAGATCTC	PNC-GBKT7
	MePRE6 -BD-R	ggtctcagcagaccacaagtCTACTCCA TAAGCAAACTC	

MebHLH149	MebHLH149-Enn-F	agtggtctctgtccagtcctATGGCTTCGTTGATCTCAAG	PNC-BiFC-Enn
	MebHLH149-Enn-R	ggtctcagcagaccacaagtGCTTGTGGGCCTAACCGG	
MePPE5	MePRE5-Ecc-F	agtggtctctgtccagtcctATGTCTAGCAGAAGGTCCAG	PNC-BiFC-Ecc
	MePRE5-Ecc-R	ggtctcagcagaccacaagtCATGATTAAACTCCTTATTATG	
MePPE6	MePRE6-Ecc-F	agtggtctctgtccagtcctATGTCTAGCAGAAGATCTC	PNC-BiFC-Ecc
	MePRE6-Ecc-R	ggtctcagcagaccacaagtCTCCATAAGCAAACTCCTA	

Table S3. Primers used for PCR detection.

Gene	Primer	Sequence	Vector
MebHLH149	PEGFP-C5	CATGGTCCTGCTGGAGTTCGTG	pNC-Green-SubN
	Nos-R	ACCGGCAACAGGATTCAATC	
MebHLH149	CsCMV-F	TGGGCGCTAATTAGTTTACTGCA	pNC-CsCMV
	CsCMV-R	GGTCAAGACGGCTCAACTCTTCA	
MebHLH149	MebHLH149-MCS35S-F	agtggtctctgtccagtcctATGGCTTCGTTGATCTCAAGC	PNC-Cam1304-MCS35S
	M13R(-48)	AGCGGATAACAATTTCACACAGGA	
MebHLH149	T7	TAATACGACTCACTATAGGG	PNC-GADT7
	3'AD	AGATGGTGCACGATGCACAG	
MePPE5/MePPE6	T7	TAATACGACTCACTATAGGG	PNC-GBKT7
	3'BD	TAAGAGTCACTTTAAAATTTGTATC	
MebHLH149	Enn-NF	TCATGGCCGACAAGCAGAAG	PNC-BiFC-Enn
	Enn-T35SR	TTATCTGGGAACTACTCACAC	
MePPE5/MePPE6	Ecc-ProF	GCATTCTACTTCTATTGCAGC	PNC-BiFC-Ecc
	Ecc-CR	GTCGCCGATGGGGGTGTTCT	

Table S4. Cassava (*Manihot esculenta*) gene IDs and protein accession numbers used this study.

Gene	Gene ID
MebHLH149	Manes.09G177600
MebHLH153	Manes.11G129300
MebHLH106	Manes.08G061000
MebHLH162	Manes.14G067000
MebHLH61	Manes.10G076700
MebHLH147-1	Manes.03G206200
MebHLH147-2	Manes.15G001200
MebHLH147-3	Manes.08G110500
MeCAD15	Manes.07G069780
MeCAD16	Manes.10G076700
MeCAD8	Manes.13G117800
MePOD12	Manes.02G182500
MeGST23	XM_043954224.1
MePPE5	A0A2C9UGZ5
MePPE6	A0A2C9WNG9
MeNCED6	XM_021752964
MeEDS1	XM_021778529.2
MeMYC2	XM_021774203.2
