# Supplementary figures and images for: Expression Pattern and Functional Analysis of MebHLH149 Gene in Response to Cassava Bacterial Blight
Source: Plants (Basel). 2024 Aug 30;13(17):2422. doi: 10.3390/plants13172422 (PMC11397265; doi:10.3390/plants13172422)

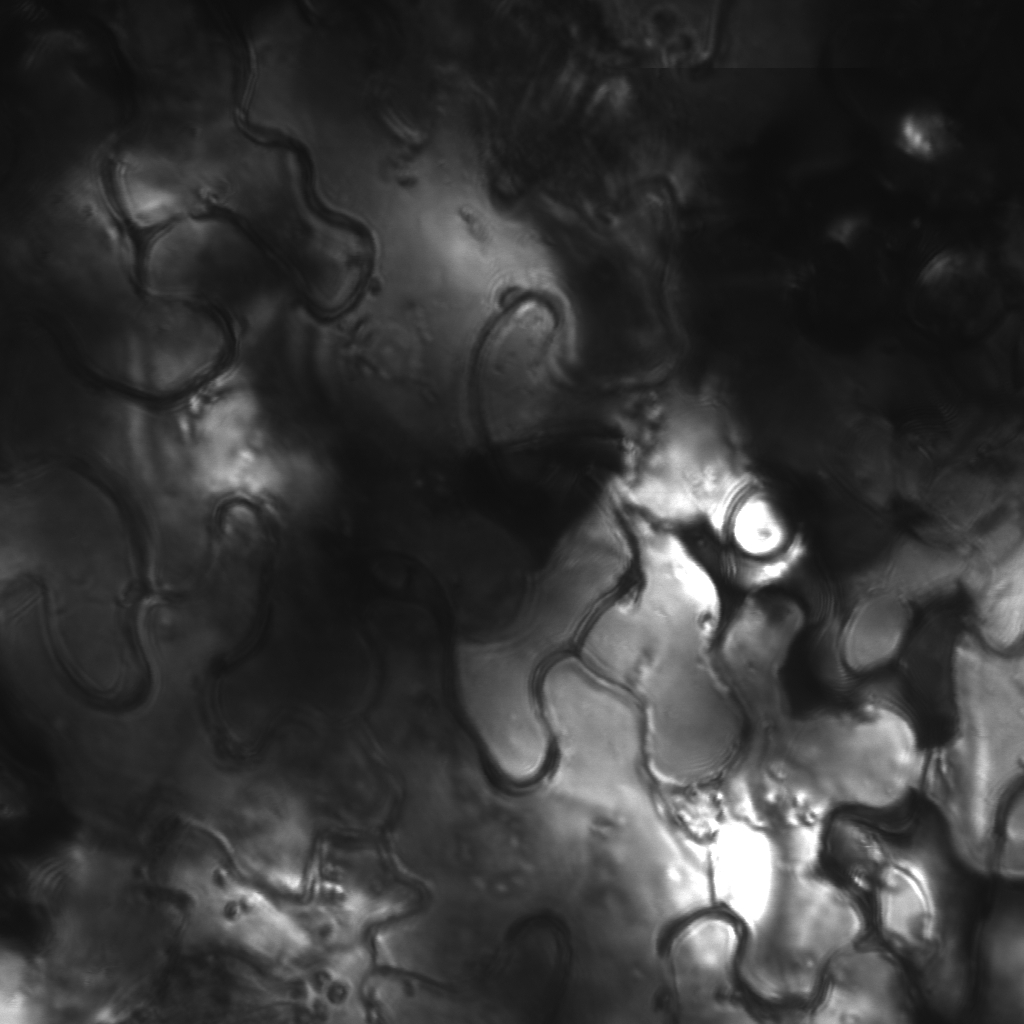

Supplement: Supplementary file 1 [file plants-13-02422-s001.zip › BIFC figures/BiFC-MebHLH149+PRE5/MebHLH149+PRE5-Bright.jpg]

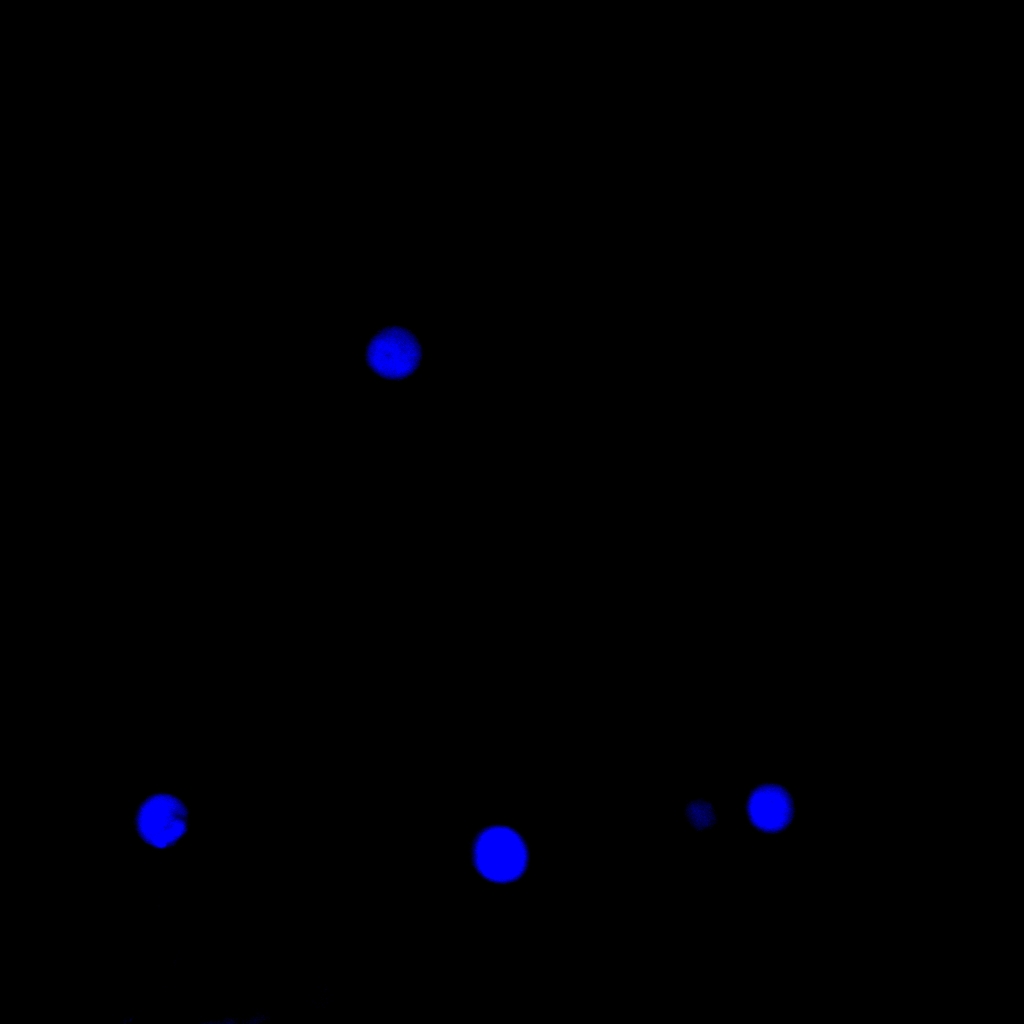

Supplement: Supplementary file 1 [file plants-13-02422-s001.zip › BIFC figures/BiFC-MebHLH149+PRE5/MebHLH149+PRE5-DAPI.jpg]

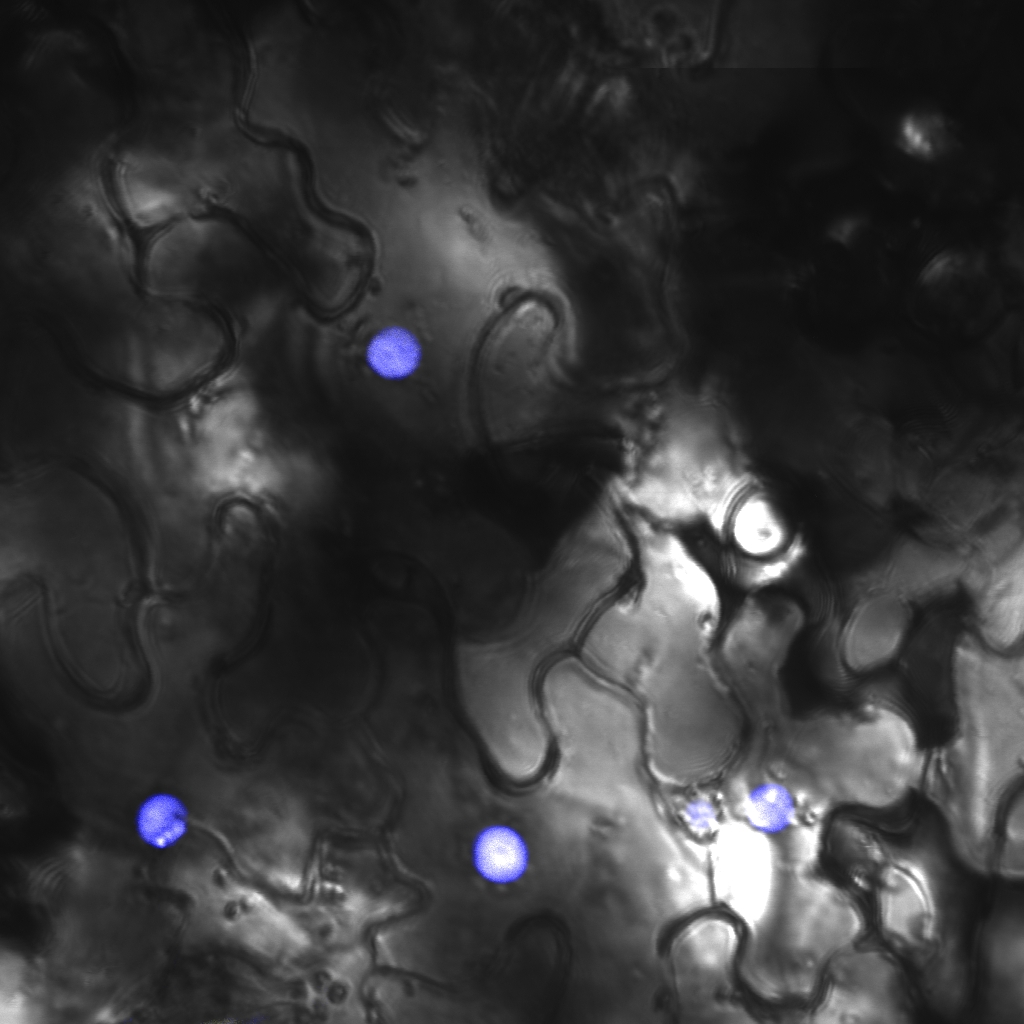

Supplement: Supplementary file 1 [file plants-13-02422-s001.zip › BIFC figures/BiFC-MebHLH149+PRE5/MebHLH149+PRE5-Megerd.jpg]

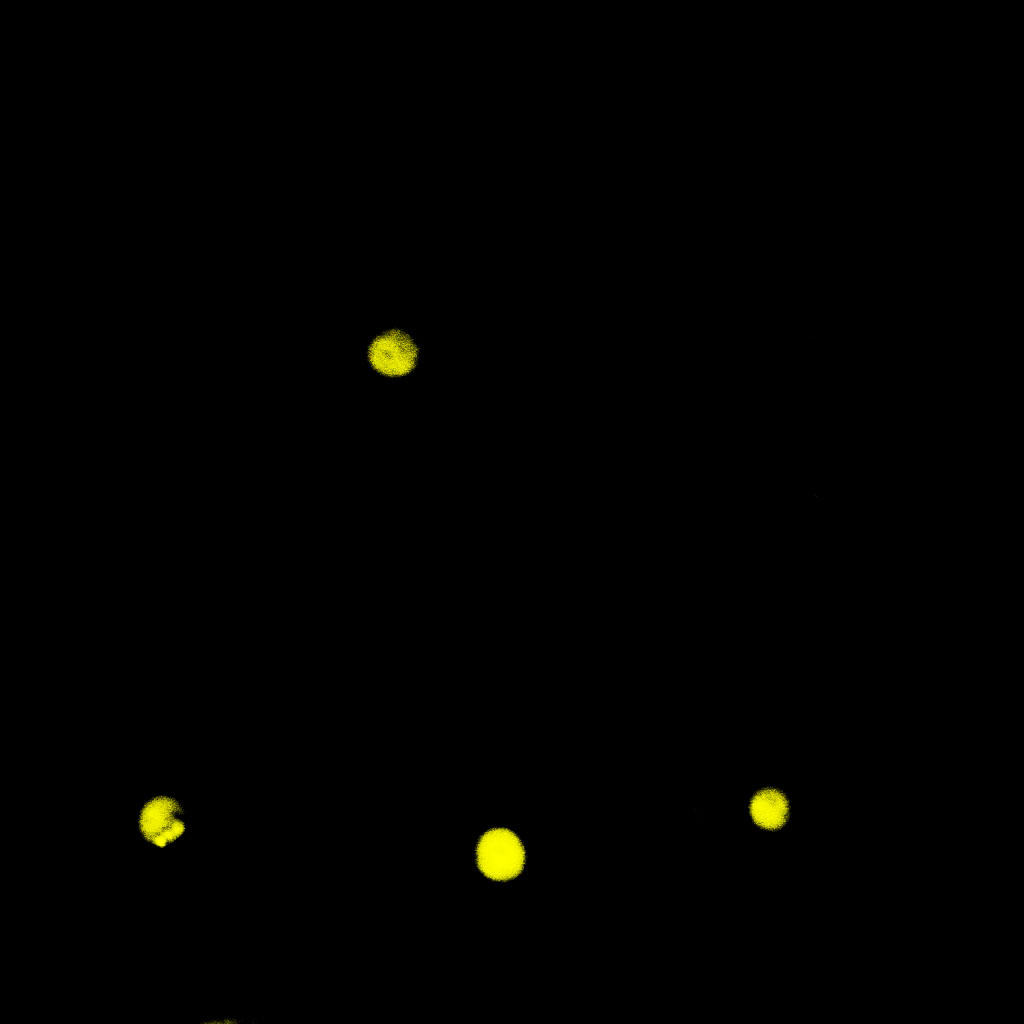

Supplement: Supplementary file 1 [file plants-13-02422-s001.zip › BIFC figures/BiFC-MebHLH149+PRE5/MebHLH149+PRE5-YFP.jpg]

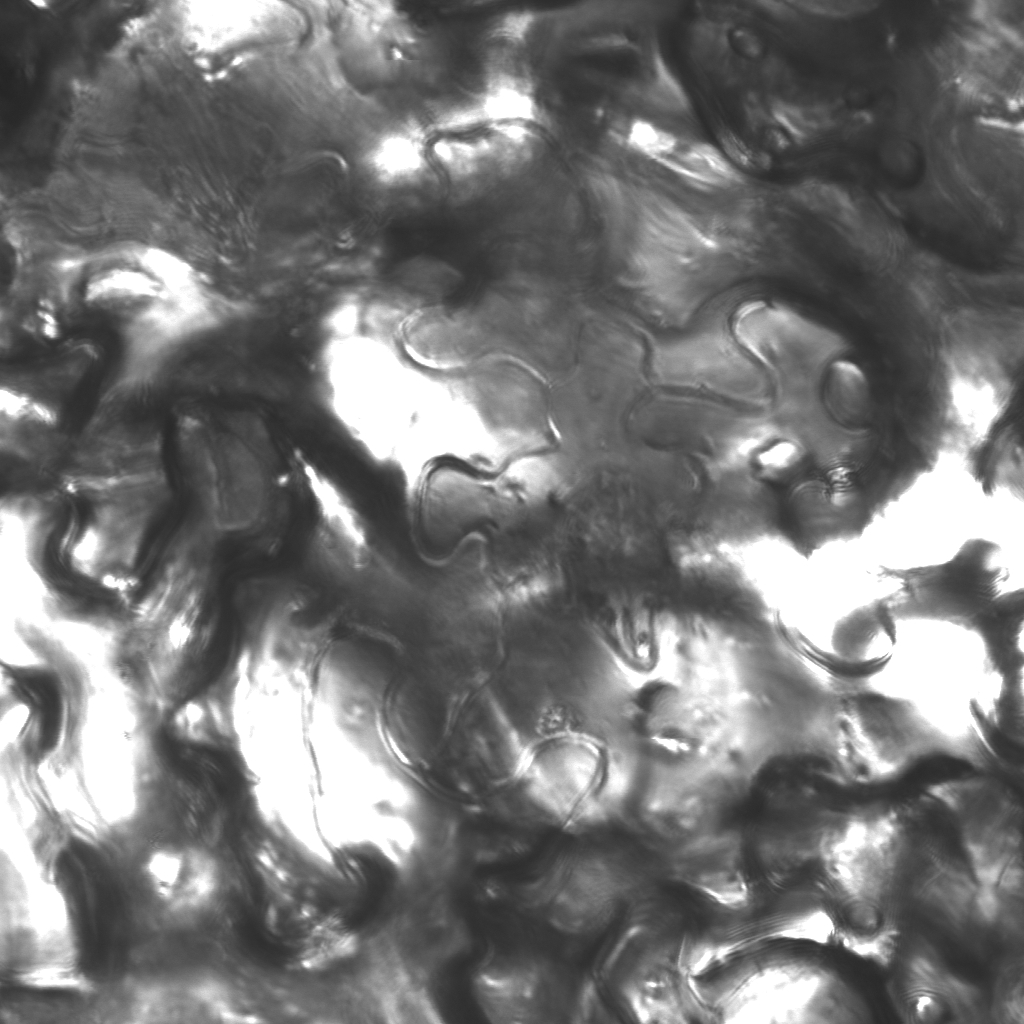

Supplement: Supplementary file 1 [file plants-13-02422-s001.zip › BIFC figures/BIFC-MebHLH149+PRE6/MebHLH149+PRE6-Bright.jpg]

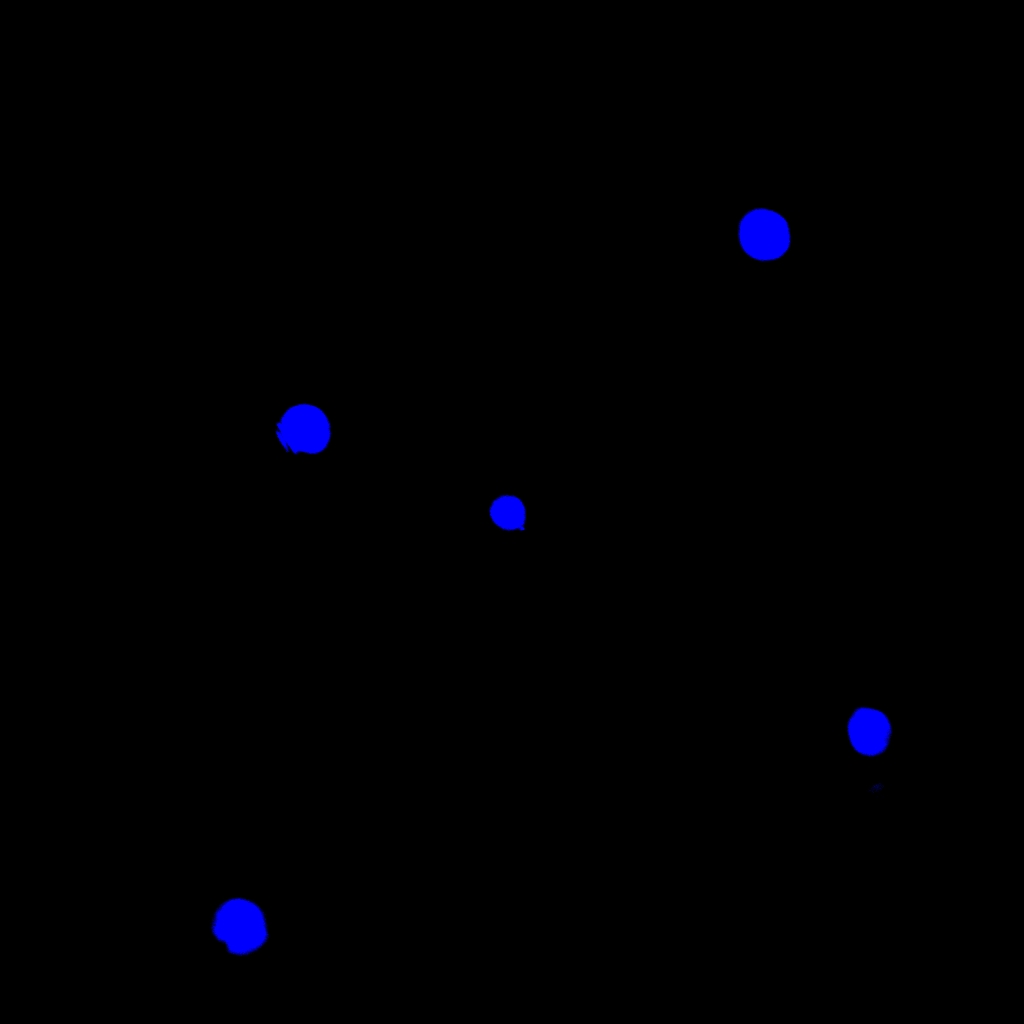

Supplement: Supplementary file 1 [file plants-13-02422-s001.zip › BIFC figures/BIFC-MebHLH149+PRE6/MebHLH149+PRE6-DAPI.jpg]

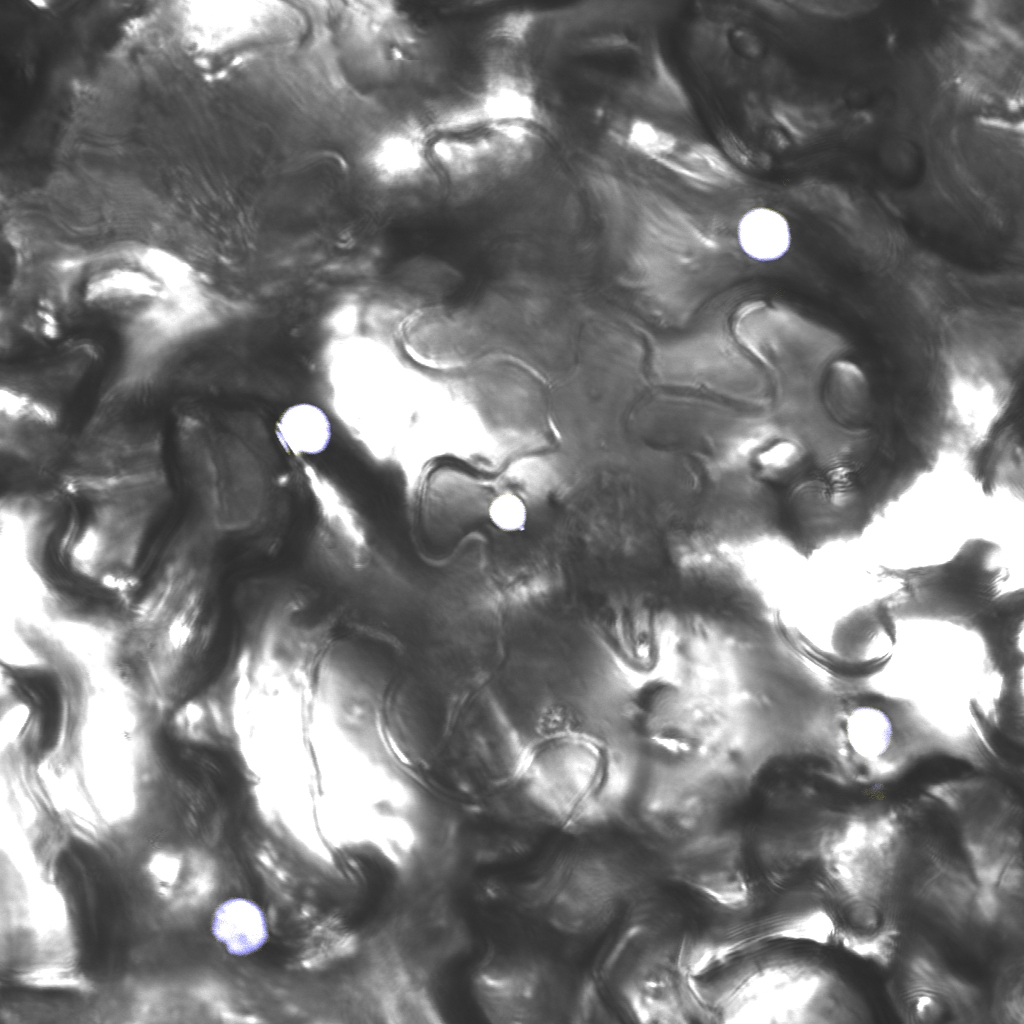

Supplement: Supplementary file 1 [file plants-13-02422-s001.zip › BIFC figures/BIFC-MebHLH149+PRE6/MebHLH149+PRE6-Merged.jpg]

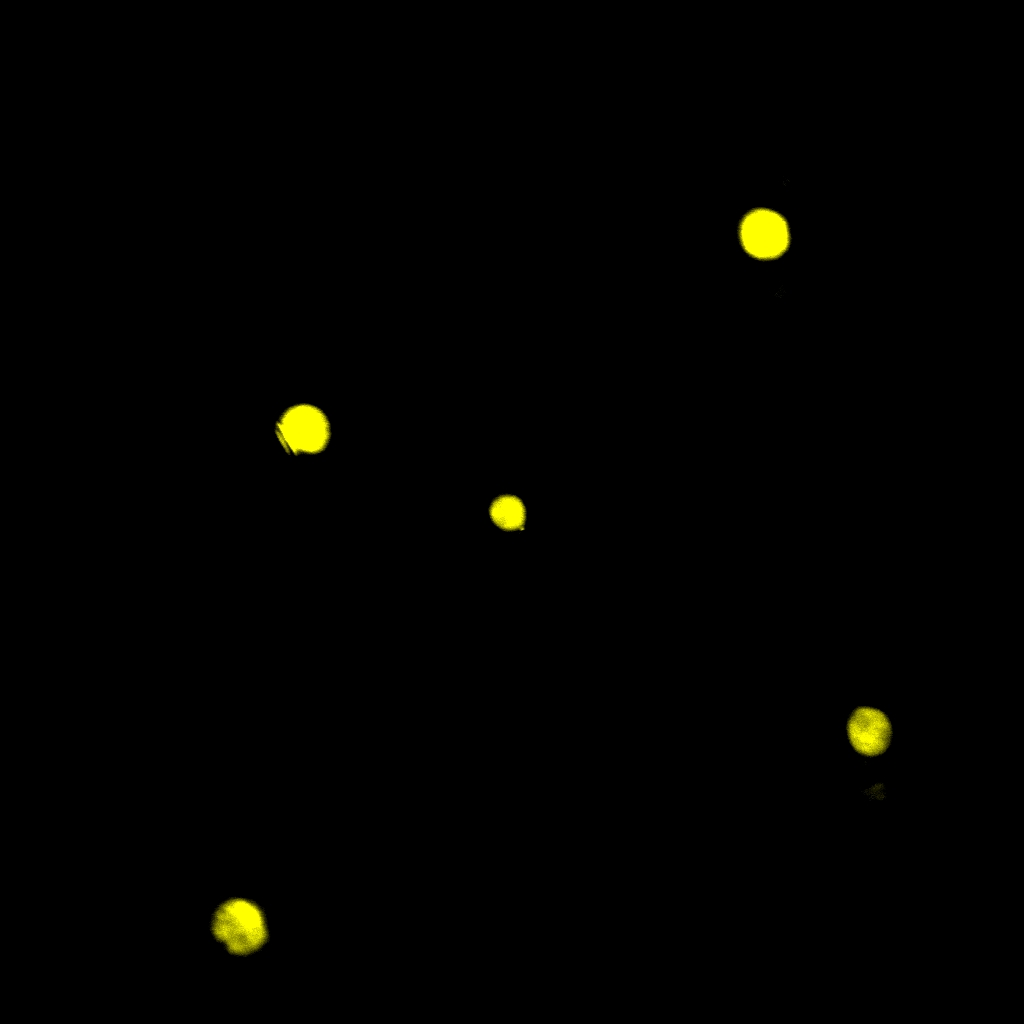

Supplement: Supplementary file 1 [file plants-13-02422-s001.zip › BIFC figures/BIFC-MebHLH149+PRE6/MebHLH149+PRE6-YFP.jpg]

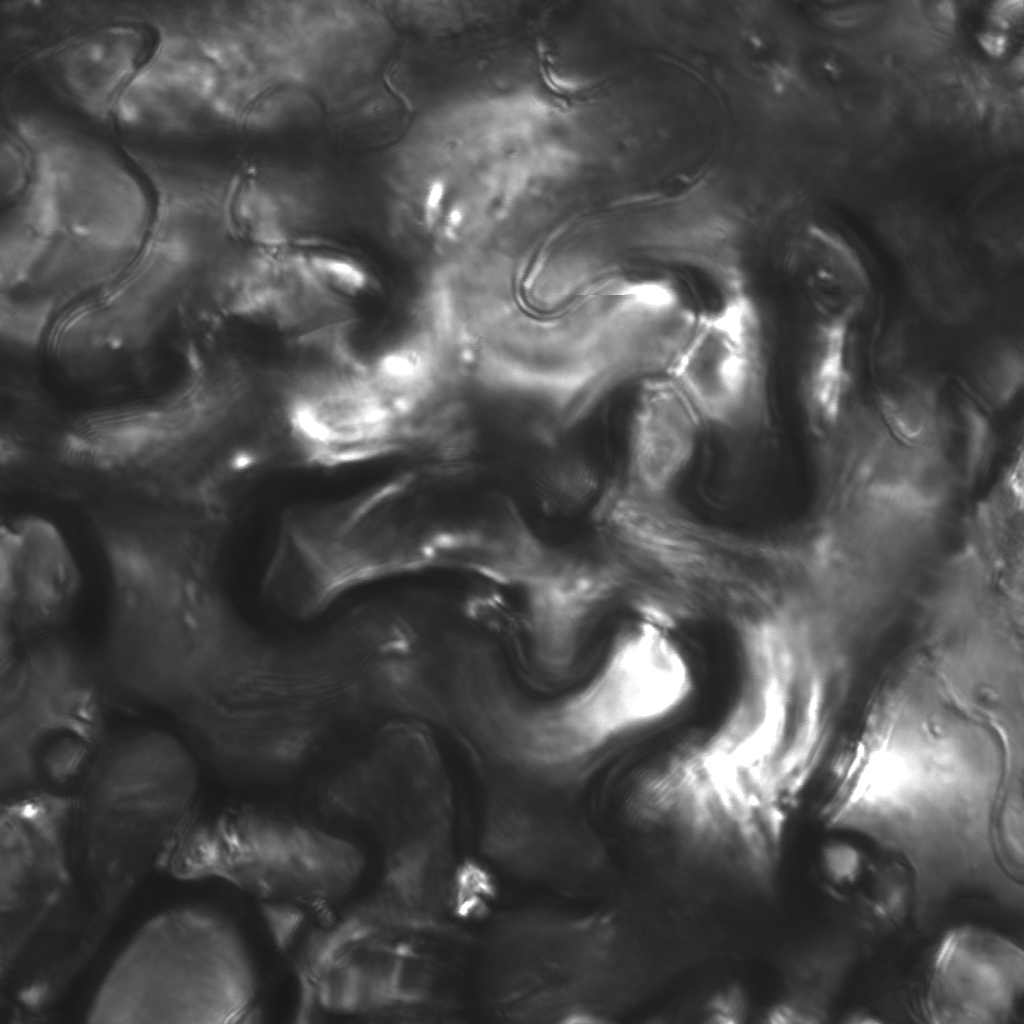

Supplement: Supplementary file 1 [file plants-13-02422-s001.zip › BIFC figures/BiFC-MebHLH149+Vector/Bright-MebHLH149+Vector.jpg]

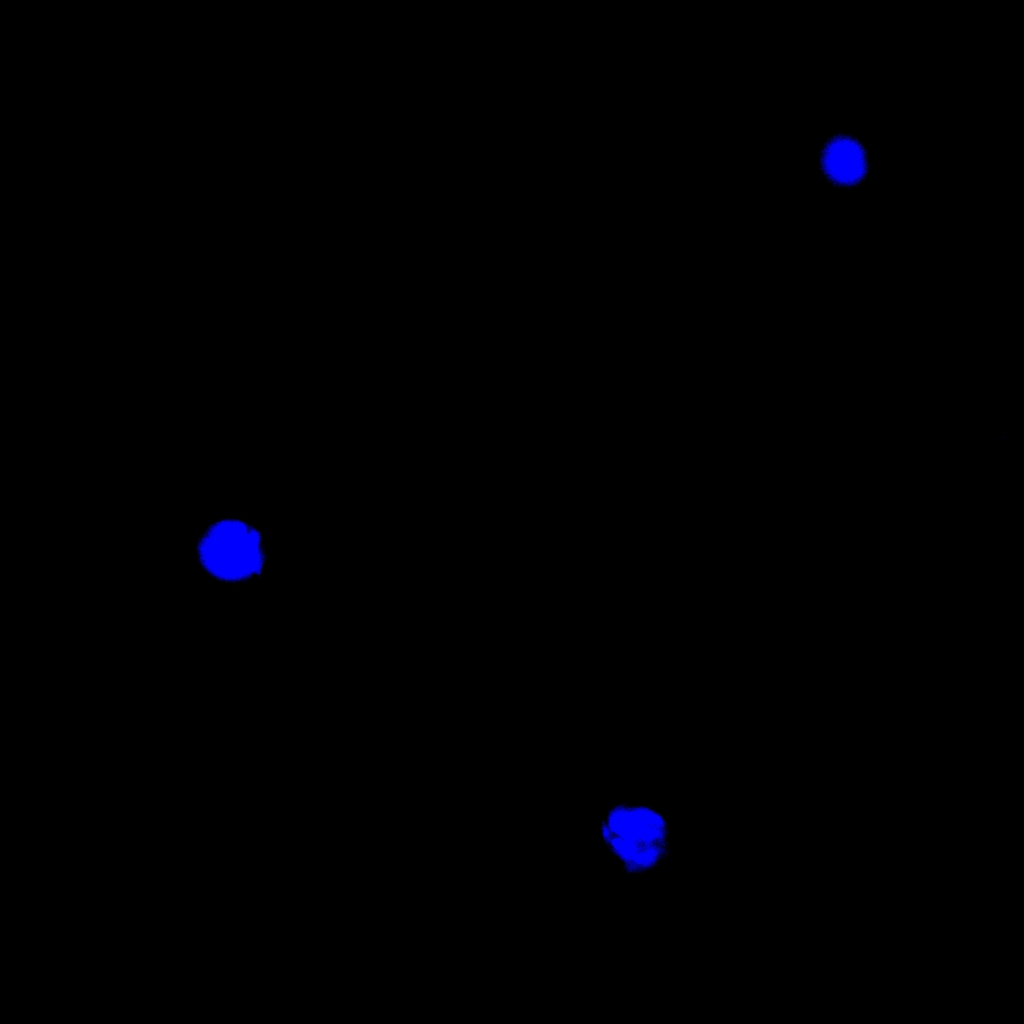

Supplement: Supplementary file 1 [file plants-13-02422-s001.zip › BIFC figures/BiFC-MebHLH149+Vector/DAPI-MebHLH149+Vector.jpg]

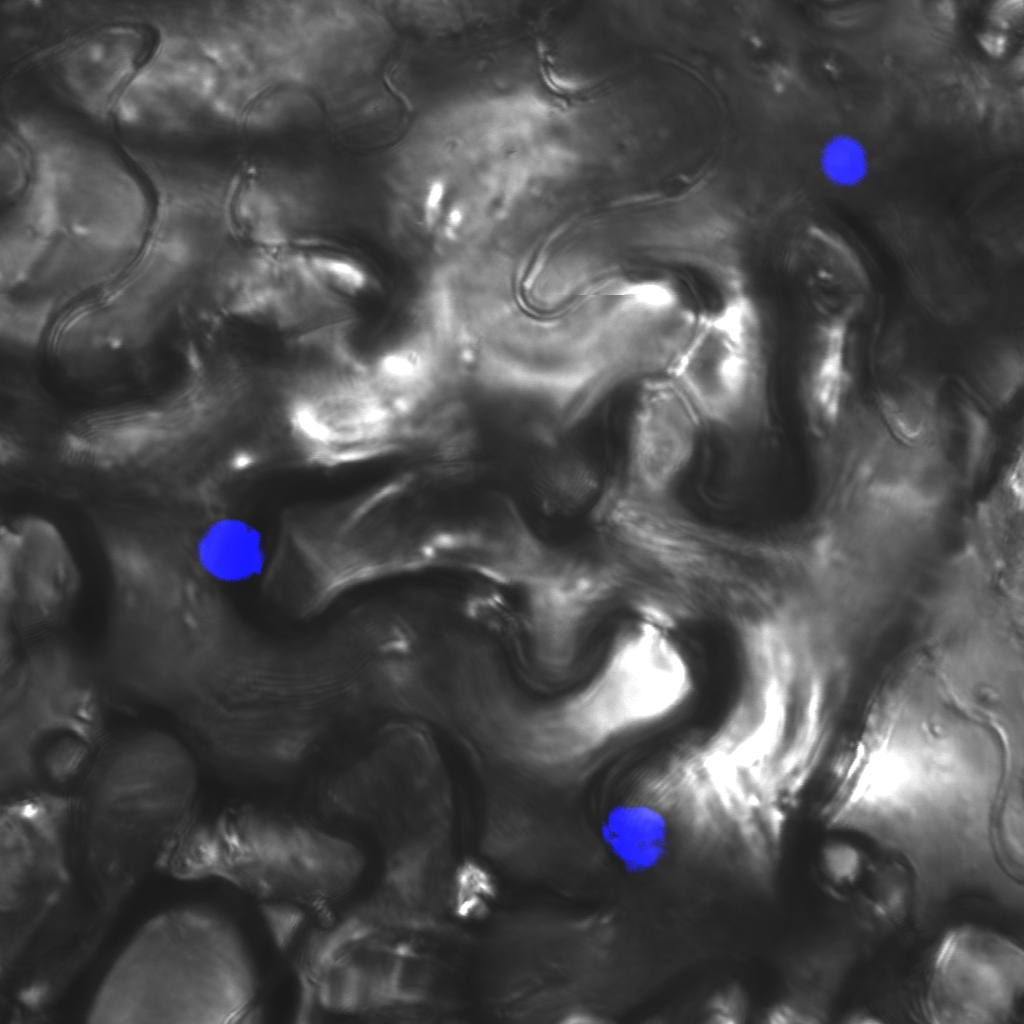

Supplement: Supplementary file 1 [file plants-13-02422-s001.zip › BIFC figures/BiFC-MebHLH149+Vector/Merged-MebHLH149+Vector.jpg]

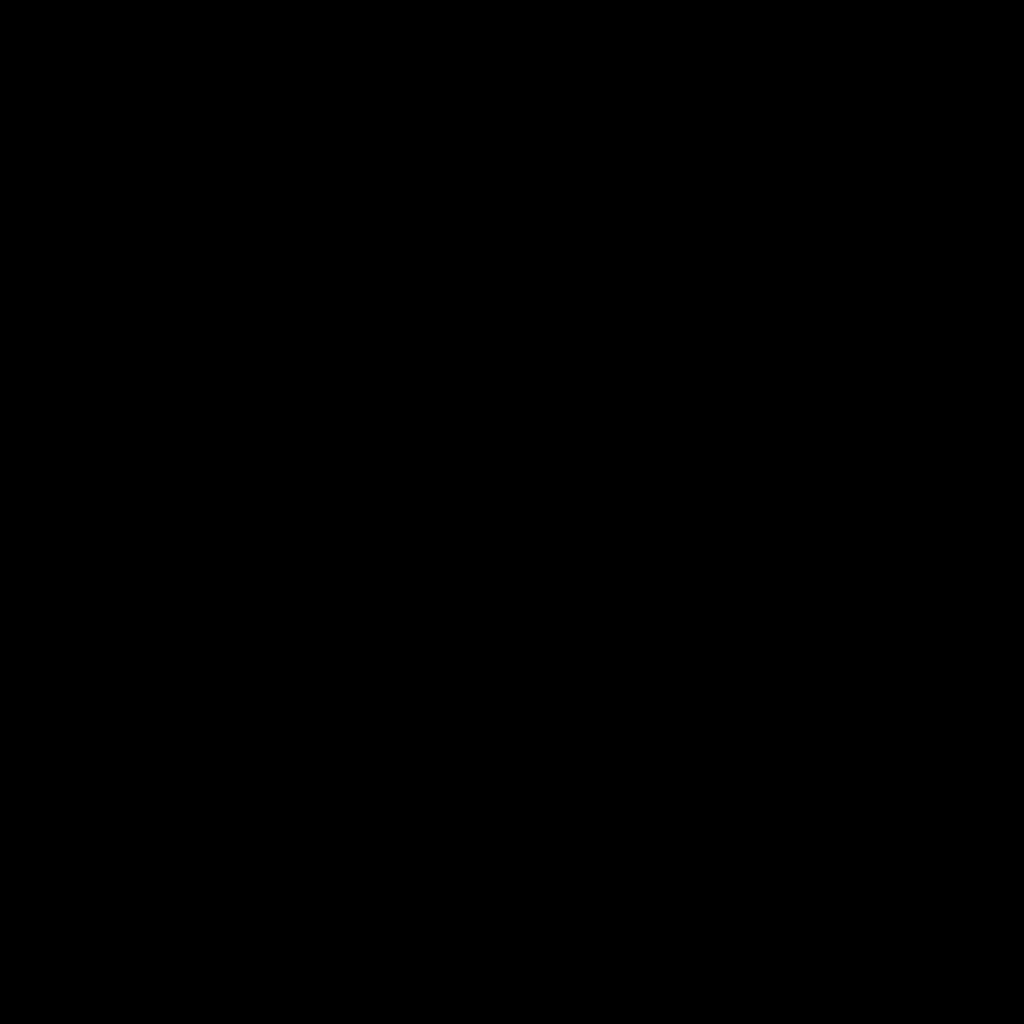

Supplement: Supplementary file 1 [file plants-13-02422-s001.zip › BIFC figures/BiFC-MebHLH149+Vector/YFP-MebHLH149+Vector.jpg]

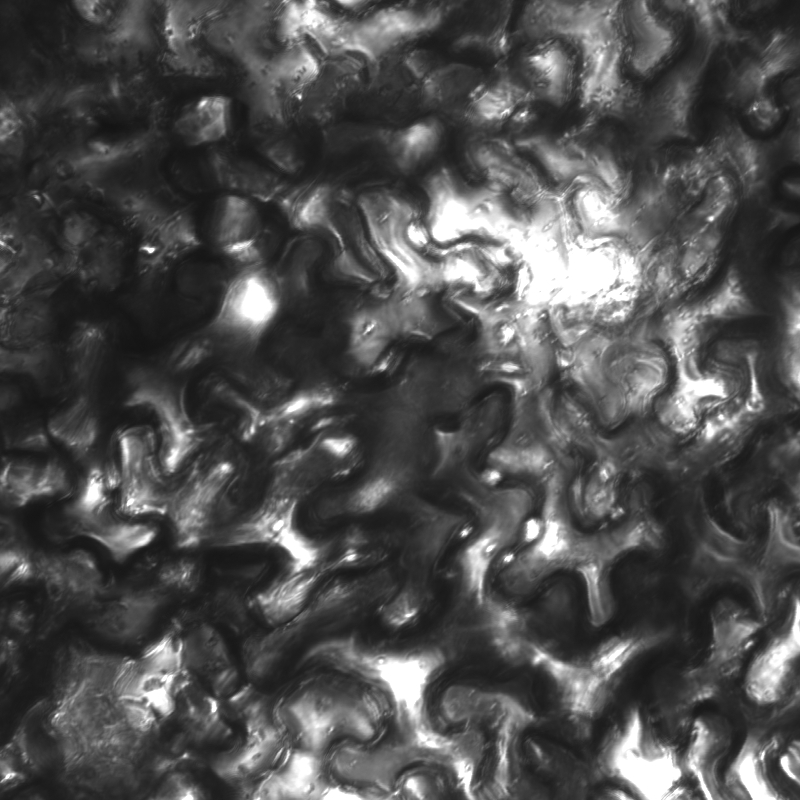

Supplement: Supplementary file 1 [file plants-13-02422-s001.zip › BIFC figures/BIFC-nYFP+cYFP/nYFP+cYFP-Bright.jpg]

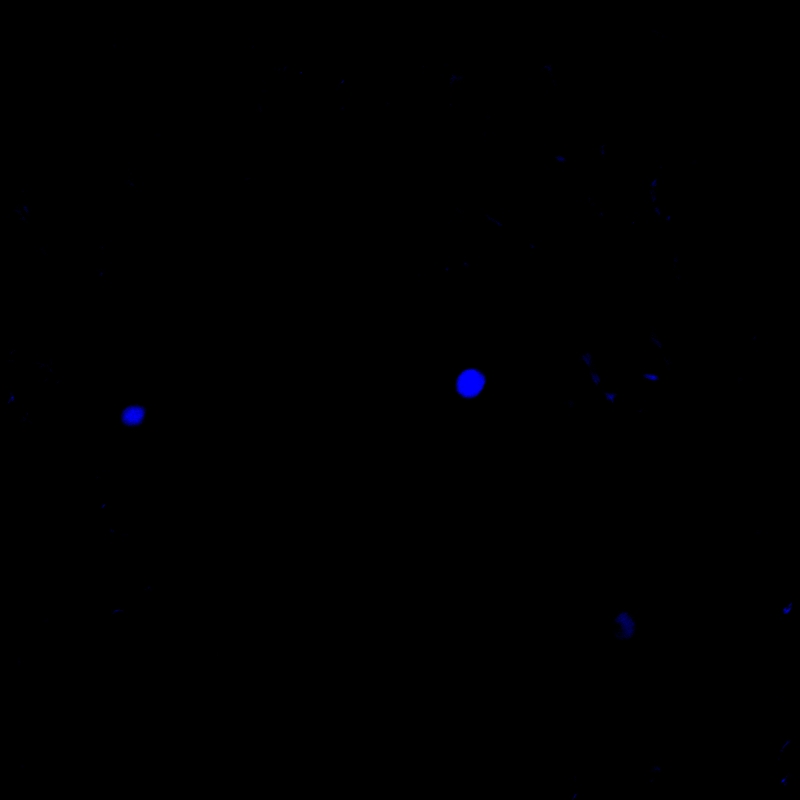

Supplement: Supplementary file 1 [file plants-13-02422-s001.zip › BIFC figures/BIFC-nYFP+cYFP/nYFP+cYFP-DAPI.jpg]

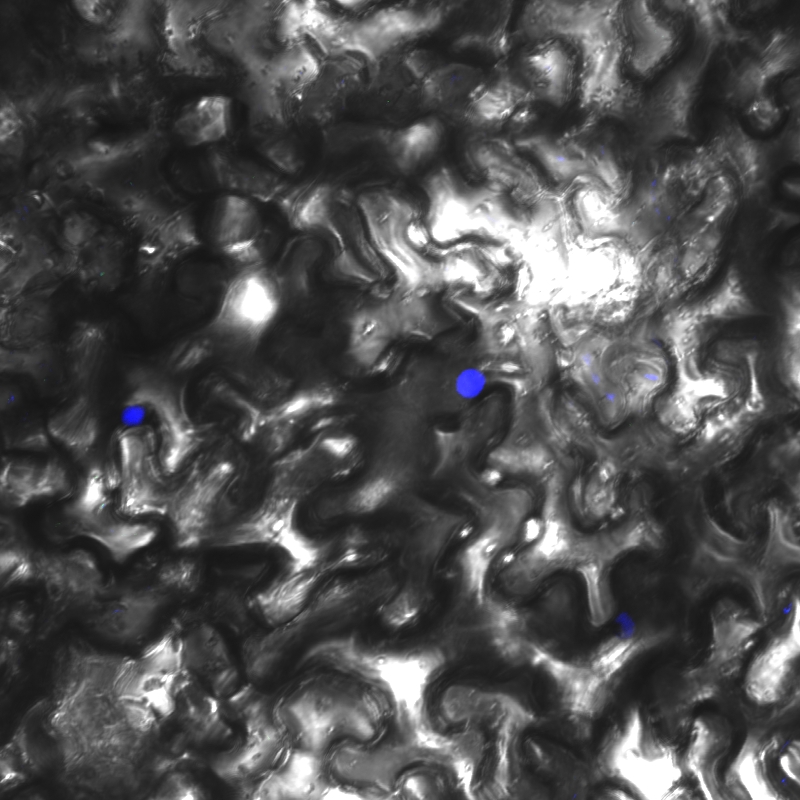

Supplement: Supplementary file 1 [file plants-13-02422-s001.zip › BIFC figures/BIFC-nYFP+cYFP/nYFP+cYFP-Merged.jpg]

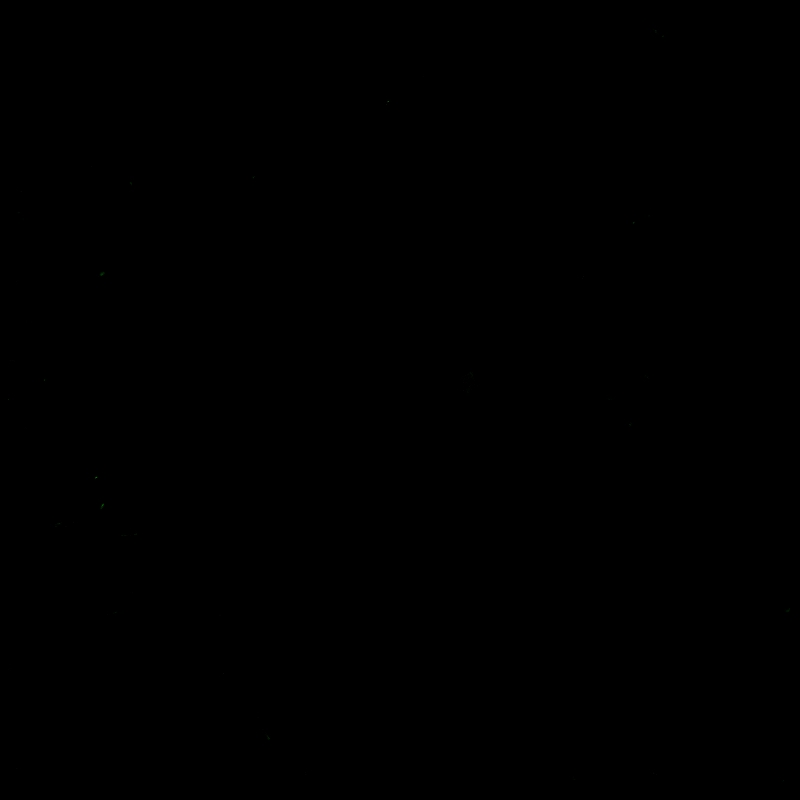

Supplement: Supplementary file 1 [file plants-13-02422-s001.zip › BIFC figures/BIFC-nYFP+cYFP/nYFP+cYFP-YFP.jpg]

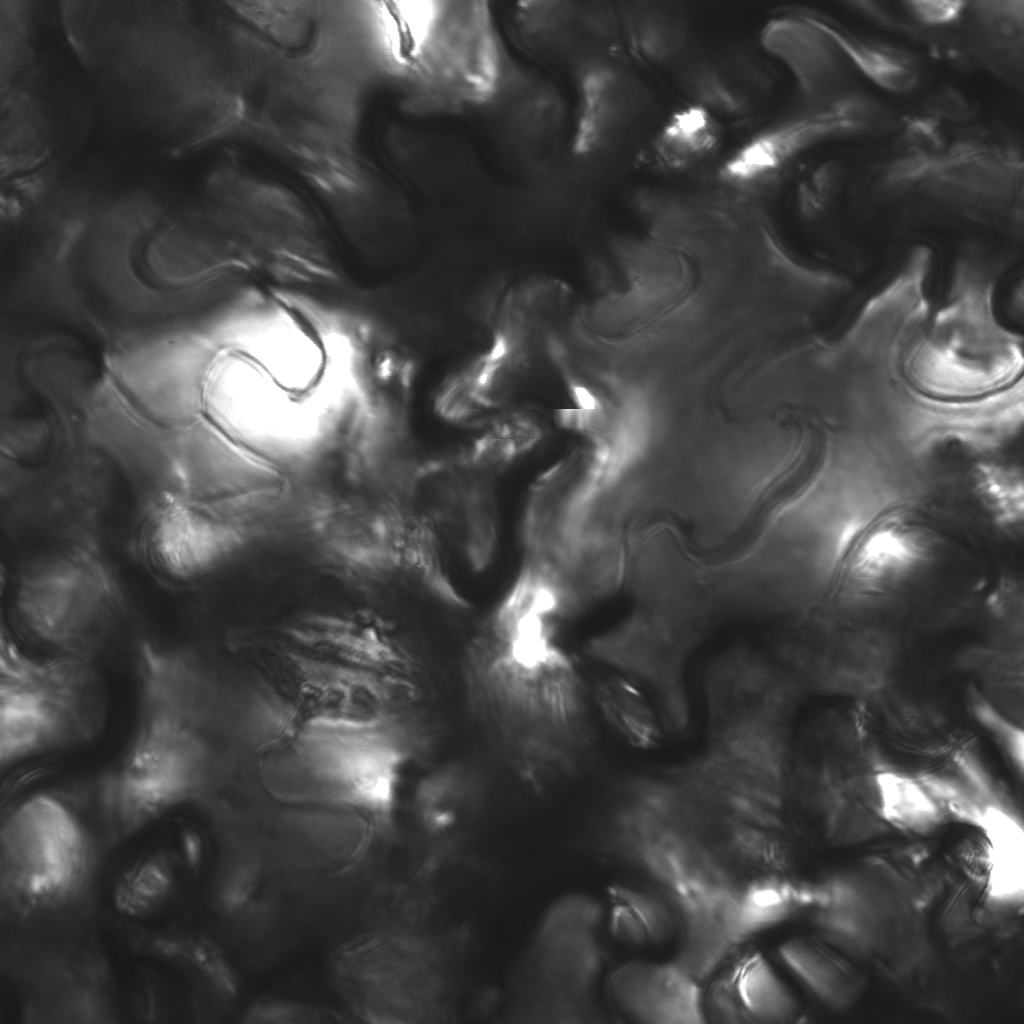

Supplement: Supplementary file 1 [file plants-13-02422-s001.zip › BIFC figures/BiFC-Vector+PRE5/Vector+PRE5-Bright.jpg]

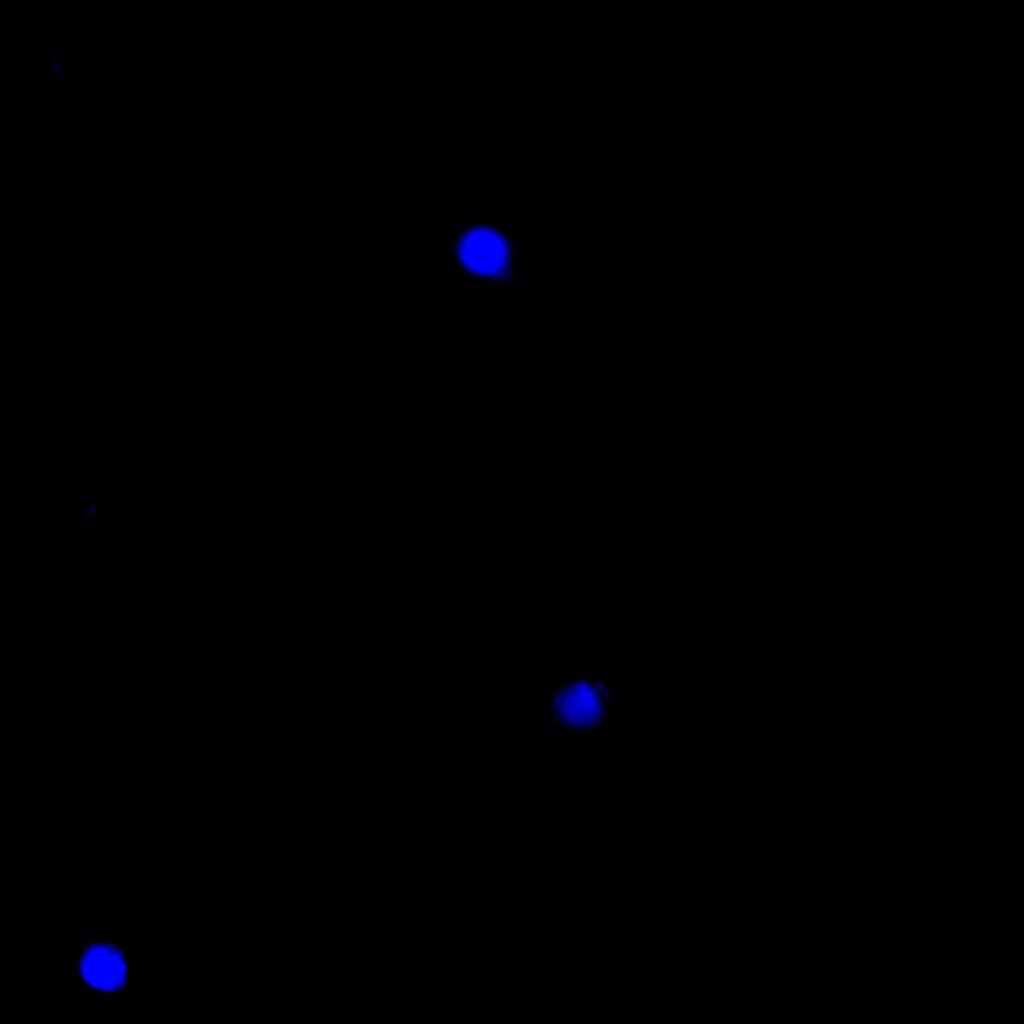

Supplement: Supplementary file 1 [file plants-13-02422-s001.zip › BIFC figures/BiFC-Vector+PRE5/Vector+PRE5-DAPI.jpg]

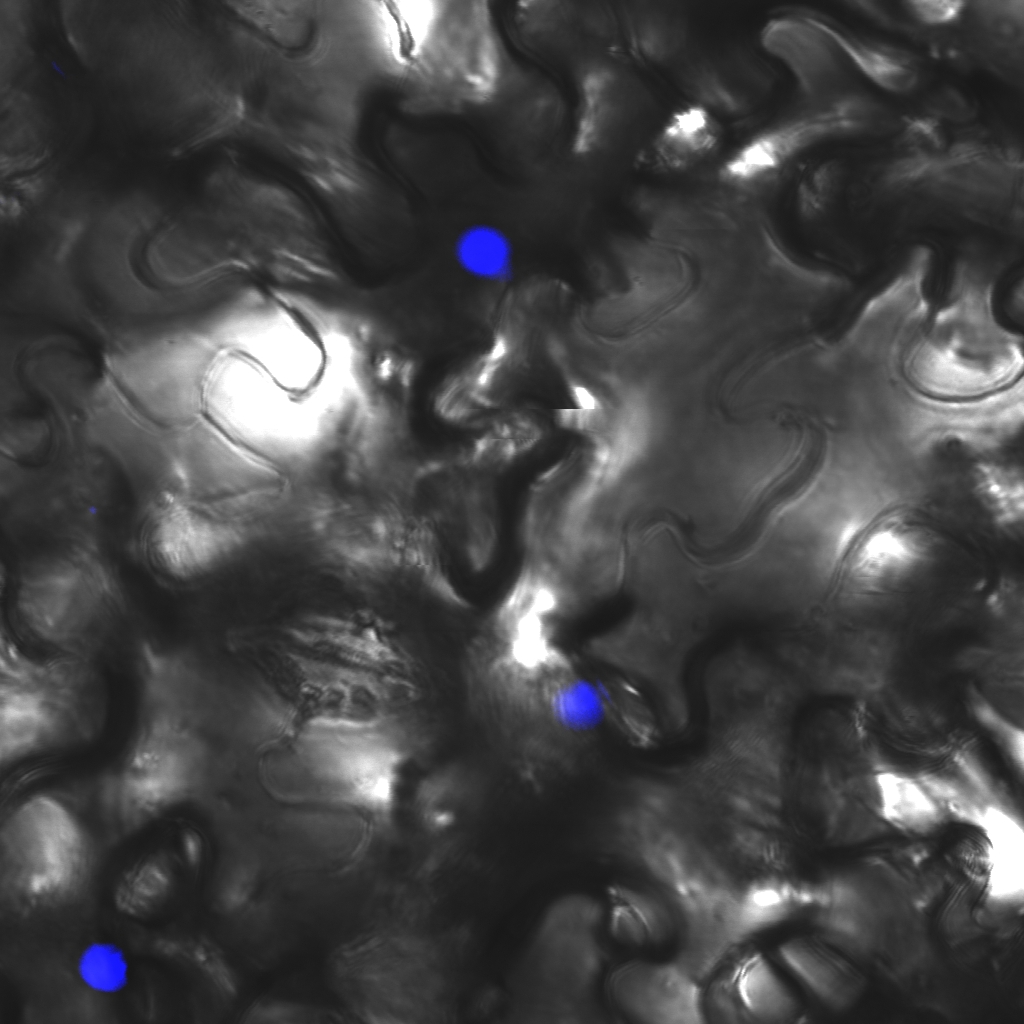

Supplement: Supplementary file 1 [file plants-13-02422-s001.zip › BIFC figures/BiFC-Vector+PRE5/Vector+PRE5-Merged.jpg]

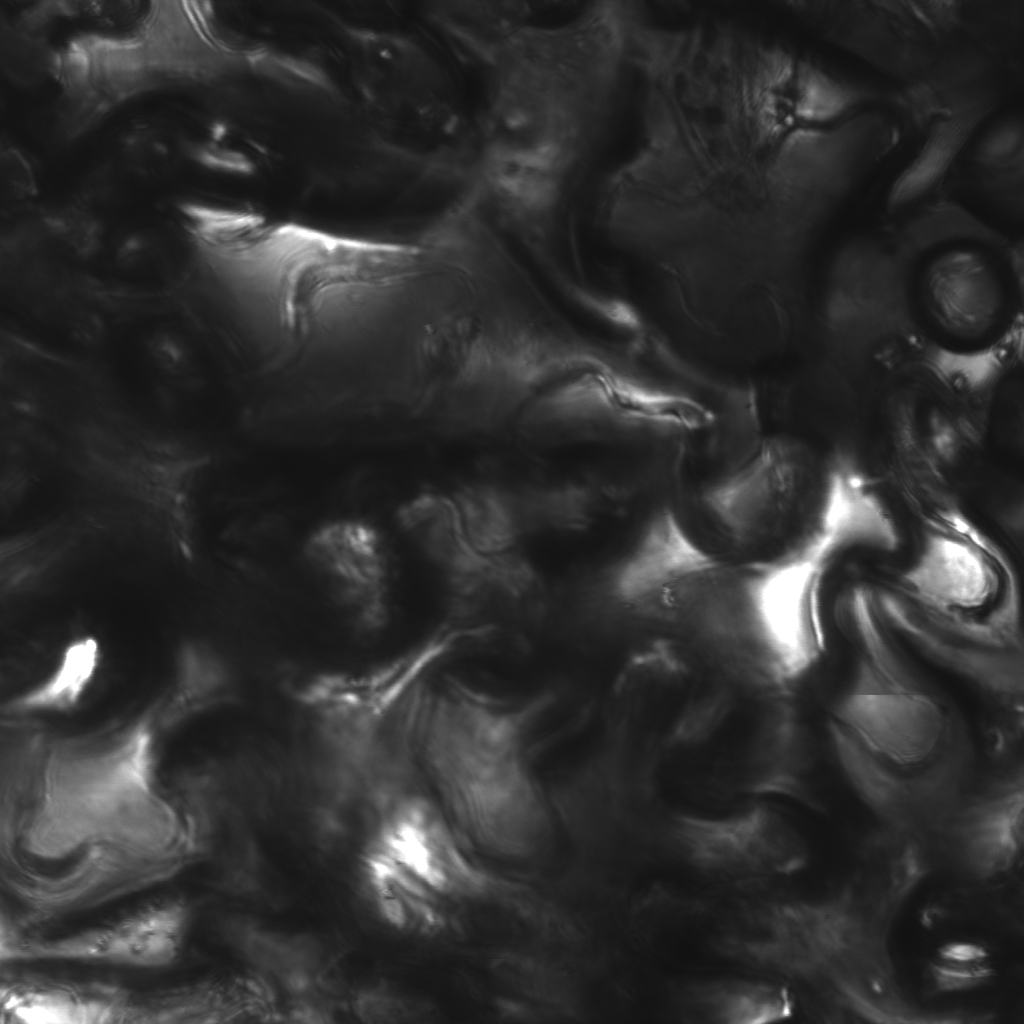

Supplement: Supplementary file 1 [file plants-13-02422-s001.zip › BIFC figures/BiFC-Vector+PRE6/Vector+PRE6-Bright.jpg]

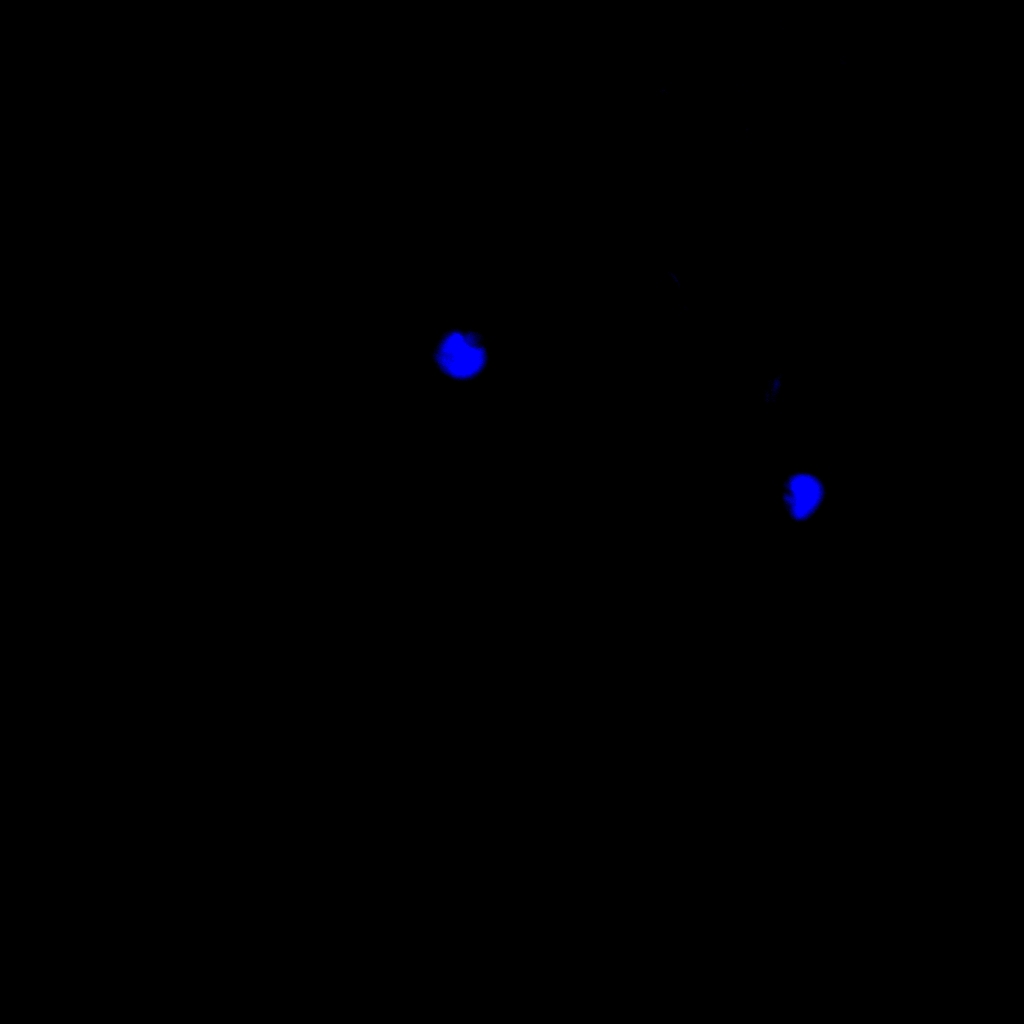

Supplement: Supplementary file 1 [file plants-13-02422-s001.zip › BIFC figures/BiFC-Vector+PRE6/Vector+PRE6-DAPI.jpg]

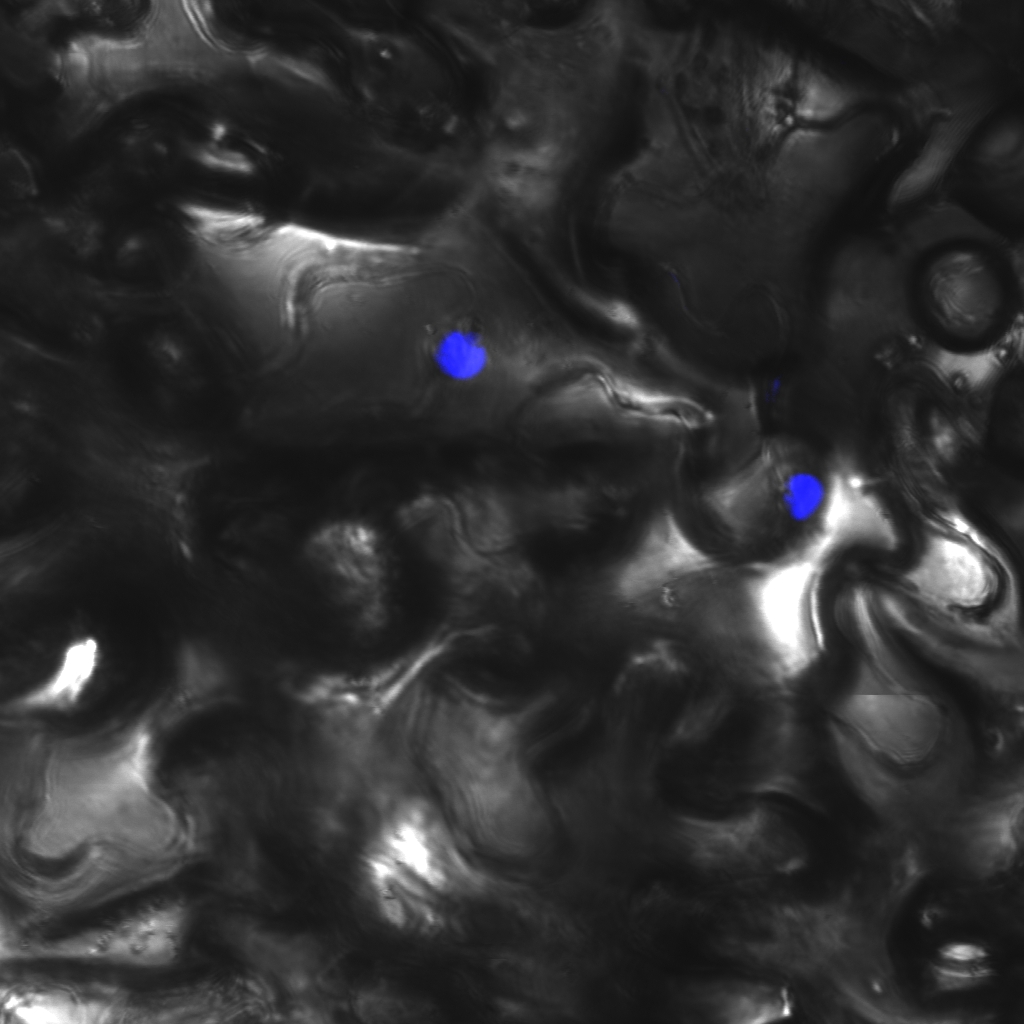

Supplement: Supplementary file 1 [file plants-13-02422-s001.zip › BIFC figures/BiFC-Vector+PRE6/Vector+PRE6-Merged.jpg]

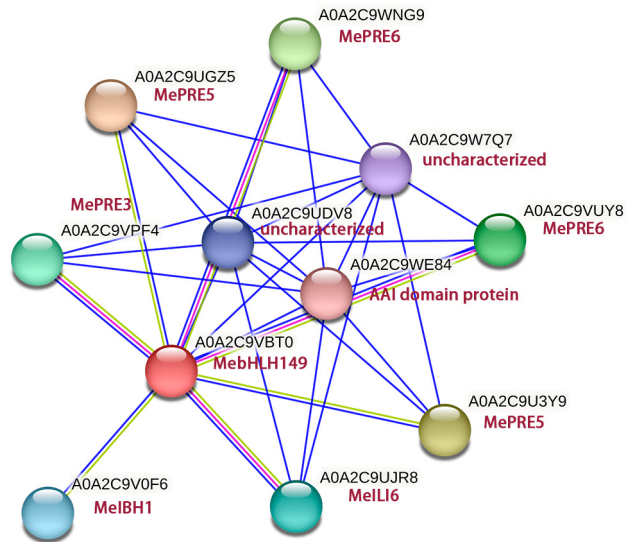

**Figure S3.** Protein-protein interaction network for MebHLH149 predicted using STRIG.

Supplement: Supplementary file 1 [file plants-13-02422-s001.zip › Figure S3.pdf]

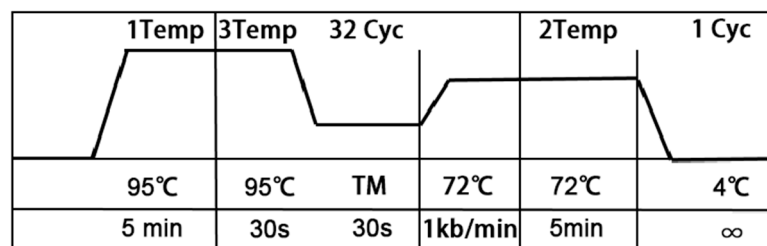

**Figure S4.** The PCR amplification reaction conditions of the cassava *bHLH149* gene.

Supplement: Supplementary file 1 [file plants-13-02422-s001.zip › Figure S4.pdf]

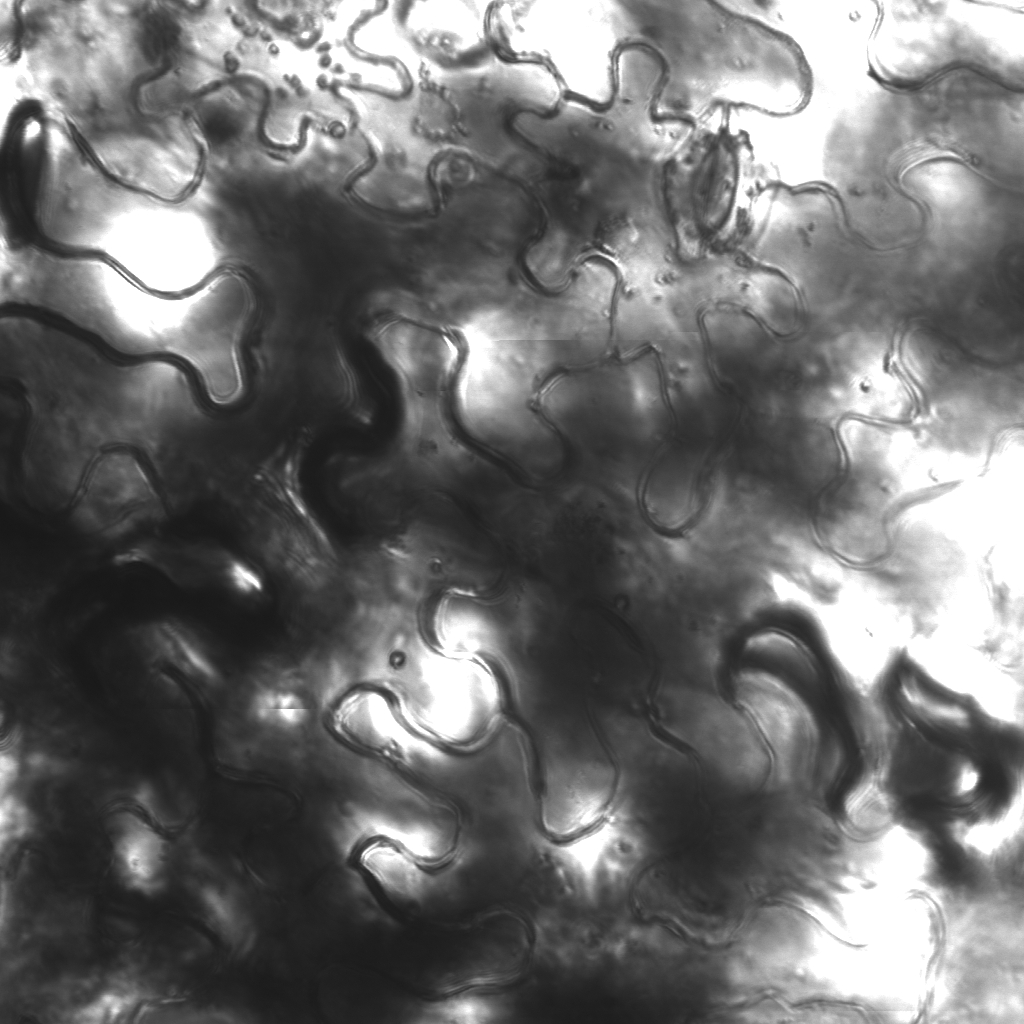

Supplement: Supplementary file 1 [file plants-13-02422-s001.zip › Subcellular localisation figures/35S-GFP.jpg.frames/35S-GFP-Bright.jpg]

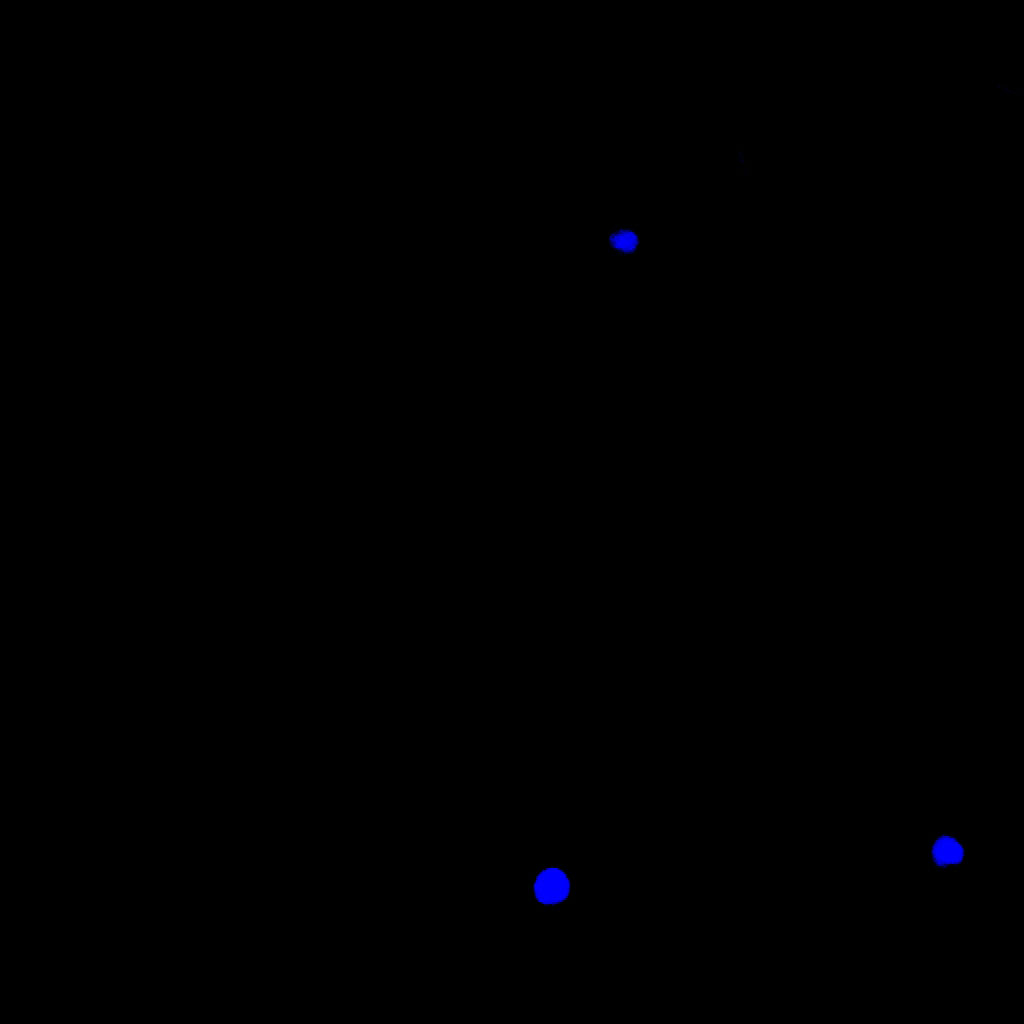

Supplement: Supplementary file 1 [file plants-13-02422-s001.zip › Subcellular localisation figures/35S-GFP.jpg.frames/35S-GFP-DAPI.jpg]

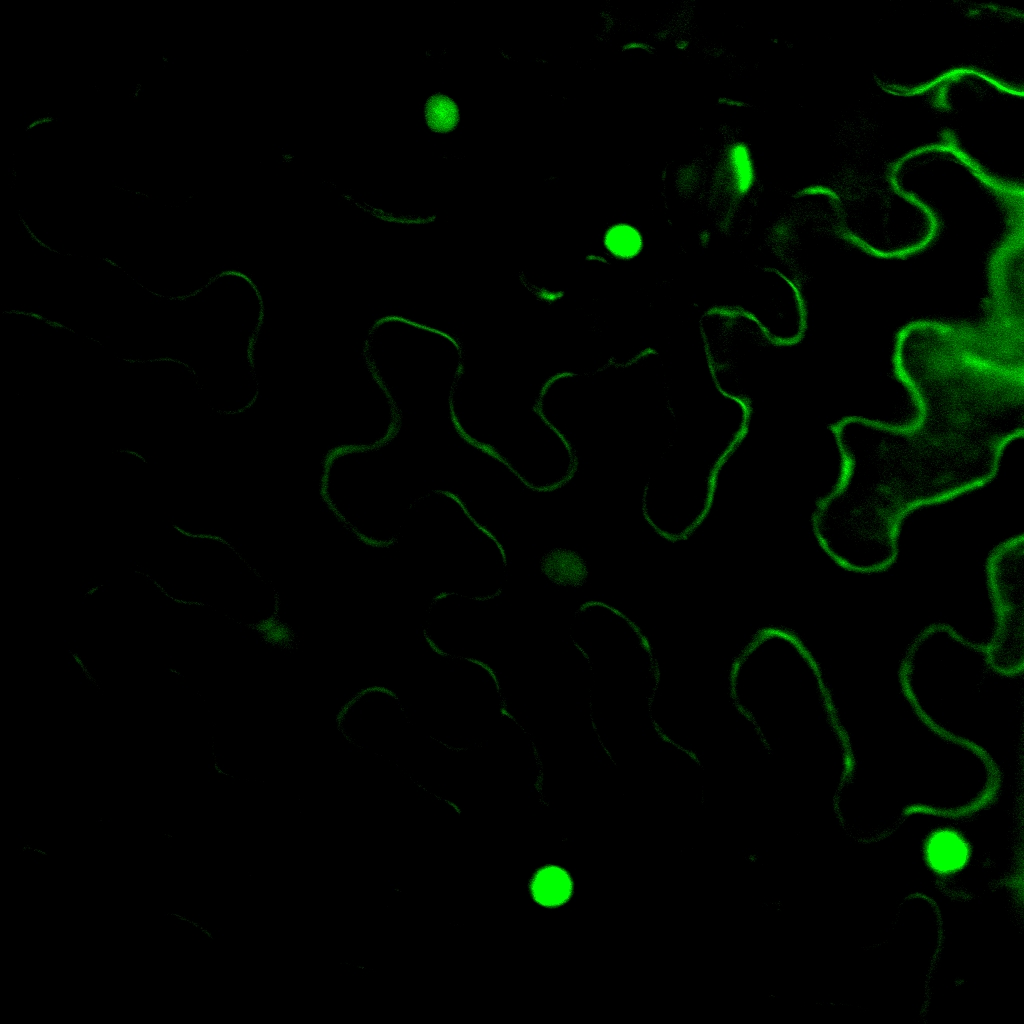

Supplement: Supplementary file 1 [file plants-13-02422-s001.zip › Subcellular localisation figures/35S-GFP.jpg.frames/35S-GFP-GFP.jpg]

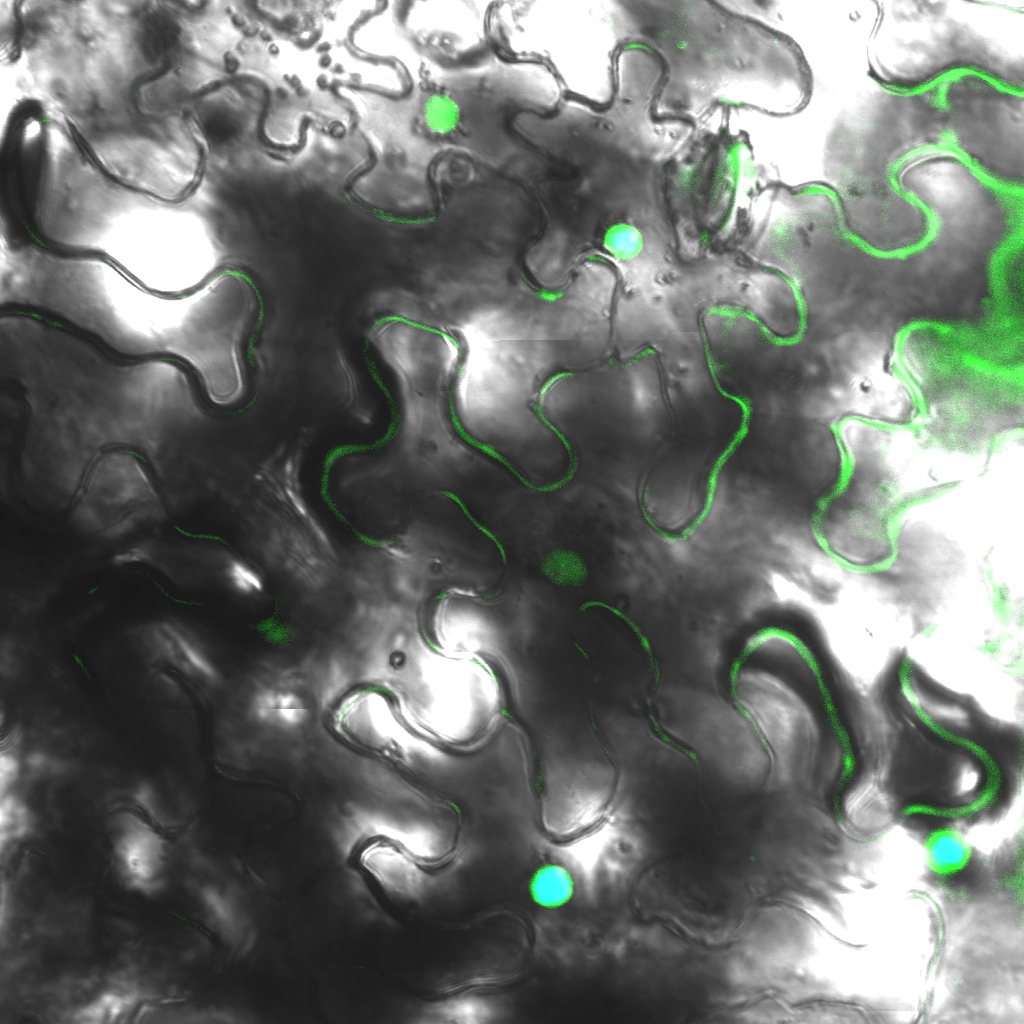

Supplement: Supplementary file 1 [file plants-13-02422-s001.zip › Subcellular localisation figures/35S-GFP.jpg.frames/35S-GFP-Merged.jpg]

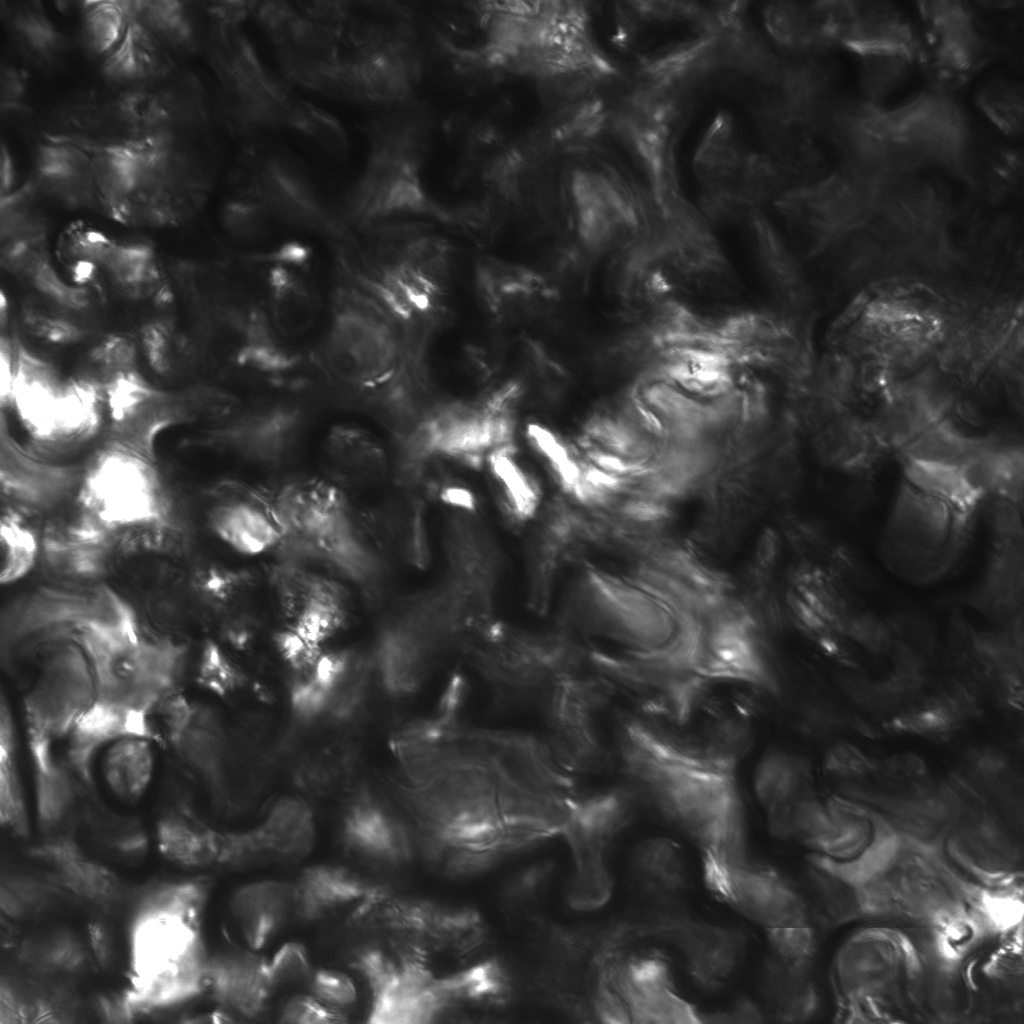

Supplement: Supplementary file 1 [file plants-13-02422-s001.zip › Subcellular localisation figures/bHLH149-35SGFP/bHLH149-35SGFP-Bright.jpg]

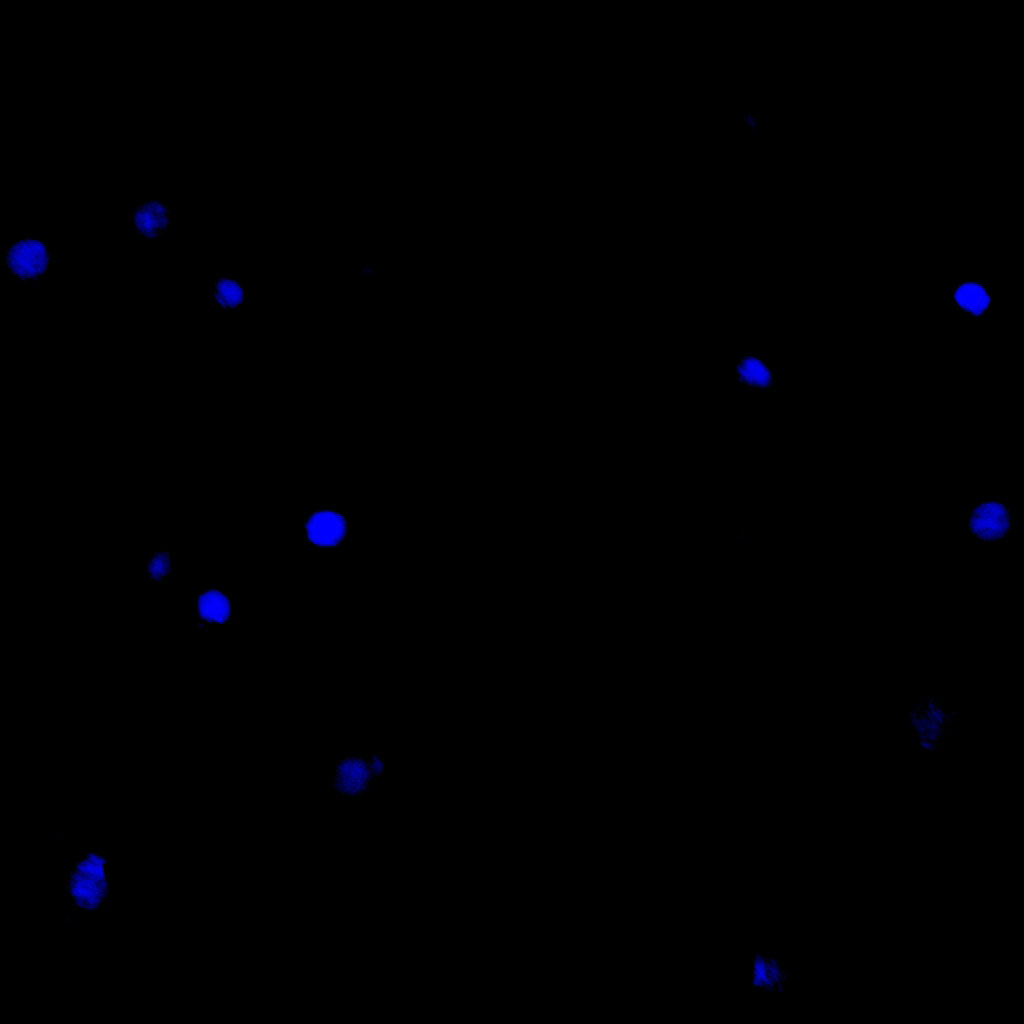

Supplement: Supplementary file 1 [file plants-13-02422-s001.zip › Subcellular localisation figures/bHLH149-35SGFP/bHLH149-35SGFP-DAPI.jpg]

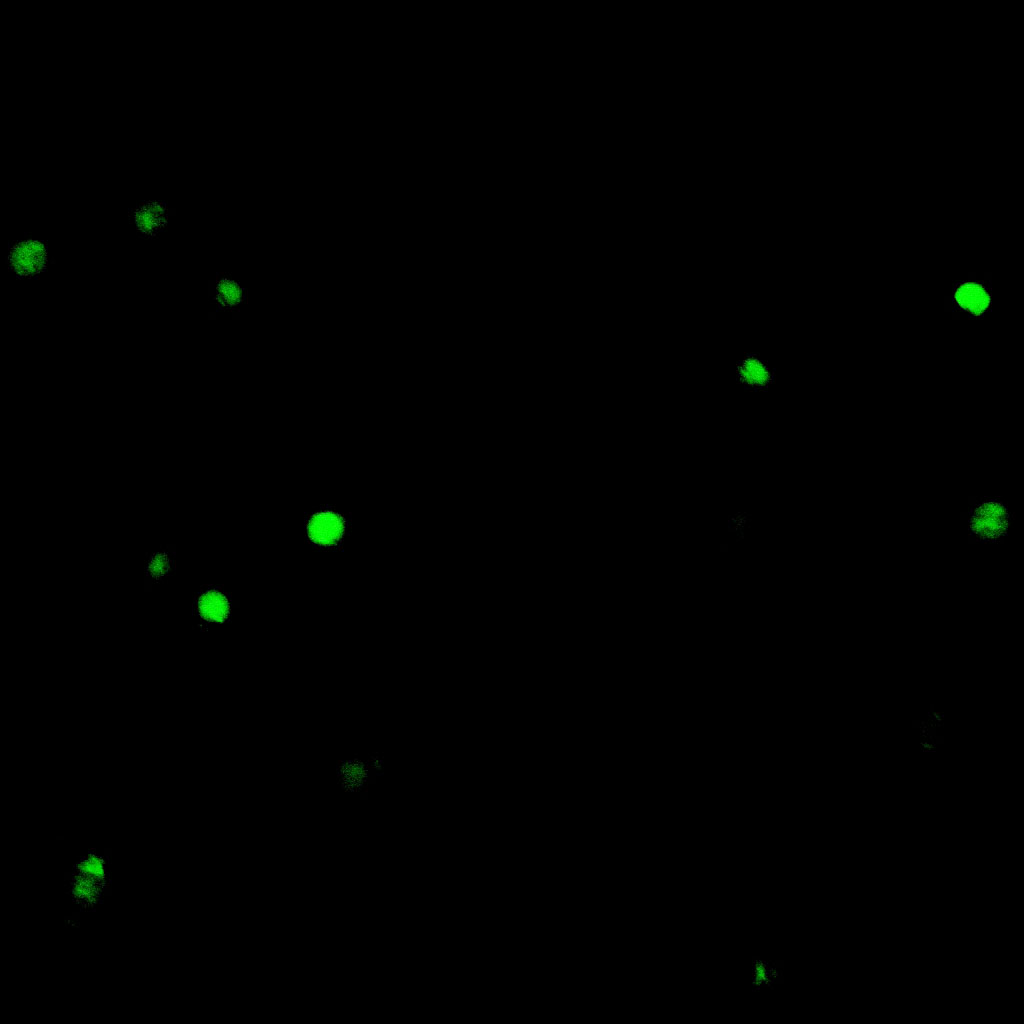

Supplement: Supplementary file 1 [file plants-13-02422-s001.zip › Subcellular localisation figures/bHLH149-35SGFP/bHLH149-35SGFP-gfp.jpg]

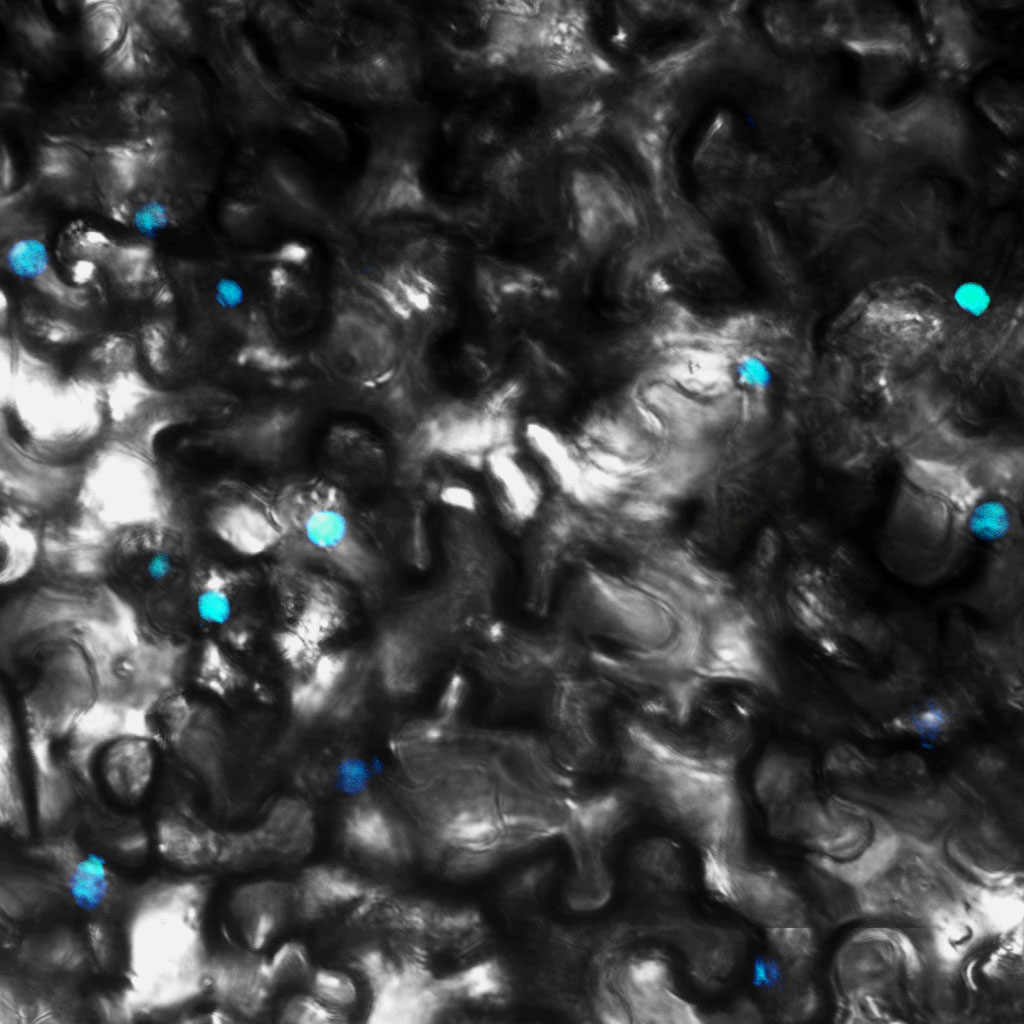

Supplement: Supplementary file 1 [file plants-13-02422-s001.zip › Subcellular localisation figures/bHLH149-35SGFP/bHLH149-35SGFP-Merged.jpg]
